# Evolutionary Mechanisms of Long-Term Genome Diversification Associated With Niche Partitioning in Marine Picocyanobacteria

**DOI:** 10.3389/fmicb.2020.567431

**Published:** 2020-09-15

**Authors:** Hugo Doré, Gregory K. Farrant, Ulysse Guyet, Julie Haguait, Florian Humily, Morgane Ratin, Frances D. Pitt, Martin Ostrowski, Christophe Six, Loraine Brillet-Guéguen, Mark Hoebeke, Antoine Bisch, Gildas Le Corguillé, Erwan Corre, Karine Labadie, Jean-Marc Aury, Patrick Wincker, Dong Han Choi, Jae Hoon Noh, Damien Eveillard, David J. Scanlan, Frédéric Partensky, Laurence Garczarek

**Affiliations:** ^1^Sorbonne Université, CNRS, UMR 7144 Adaptation and Diversity in the Marine Environment (AD2M), Station Biologique de Roscoff (SBR), Roscoff, France; ^2^LS2N, UMR CNRS 6004, IMT Atlantique, ECN, Université de Nantes, Nantes, France; ^3^School of Life Sciences, University of Warwick, Coventry, United Kingdom; ^4^CNRS, FR 2424, ABiMS Platform, Station Biologique de Roscoff (SBR), Roscoff, France; ^5^Sorbonne Université, CNRS, UMR 8227, Integrative Biology of Marine Models (LBI2M), Station Biologique de Roscoff (SBR), Roscoff, France; ^6^Genoscope, Institut de Biologie François-Jacob, Commissariat à l’Energie Atomique (CEA), Université Paris-Saclay, Évry, France; ^7^Génomique Métabolique, Genoscope, Institut de Biologie François Jacob, CEA, CNRS, Université d’Evry, Université Paris-Saclay, Évry, France; ^8^Marine Ecosystem Research Center, Korea Institute of Ocean Science and Technology, Busan, South Korea; ^9^Ocean Science and Technology School, Korea Maritime and Ocean University, Busan, South Korea; ^10^Department of Marine Biology, Korea University of Science and Technology, Daejeon, South Korea; ^11^Research Federation (FR2022) Tara Océans GO-SEE, Paris, France

**Keywords:** marine cyanobacteria, *Prochlorococcus*, *Synechococcus*, comparative genomics, niche adaptation, amino-acid substitutions, genomic islands, evolution

## Abstract

Marine picocyanobacteria of the genera *Prochlorococcus* and *Synechococcus* are the most abundant photosynthetic organisms on Earth, an ecological success thought to be linked to the differential partitioning of distinct ecotypes into specific ecological niches. However, the underlying processes that governed the diversification of these microorganisms and the appearance of niche-related phenotypic traits are just starting to be elucidated. Here, by comparing 81 genomes, including 34 new *Synechococcus*, we explored the evolutionary processes that shaped the genomic diversity of picocyanobacteria. Time-calibration of a core-protein tree showed that gene gain/loss occurred at an unexpectedly low rate between the different lineages, with for instance 5.6 genes gained per million years (My) for the major *Synechococcus* lineage (sub-cluster 5.1), among which only 0.71/My have been fixed in the long term. Gene content comparisons revealed a number of candidates involved in nutrient adaptation, a large proportion of which are located in genomic islands shared between either closely or more distantly related strains, as identified using an original network construction approach. Interestingly, strains representative of the different ecotypes co-occurring in phosphorus-depleted waters (*Synechococcus* clades III, WPC1, and sub-cluster 5.3) were shown to display different adaptation strategies to this limitation. In contrast, we found few genes potentially involved in adaptation to temperature when comparing cold and warm thermotypes. Indeed, comparison of core protein sequences highlighted variants specific to cold thermotypes, notably involved in carotenoid biosynthesis and the oxidative stress response, revealing that long-term adaptation to thermal niches relies on amino acid substitutions rather than on gene content variation. Altogether, this study not only deciphers the respective roles of gene gains/losses and sequence variation but also uncovers numerous gene candidates likely involved in niche partitioning of two key members of the marine phytoplankton.

## Introduction

Understanding how phytoplankton species have adapted to the marine environment, a dynamic system through time and space, is a significant challenge, notably in the context of rapid global change (Edwards and Richardson, 2004; Sears and Angilletta, 2011; Irwin et al., 2015; Doblin and Van Sebille, 2016). Even though these microorganisms might adapt more rapidly than larger organisms to environmental change due to their short generation times and large population sizes, the underlying mechanisms and timescales required for such evolutionary processes to occur remain mostly unknown. One of the best ways to better understand these processes is by deciphering the links between current genomic diversity and niche occupancy of these organisms. Such an approach requires complete genomes with representatives of distinct ecological niches, a resource which remains limited even with the advent of high-throughput sequencing and the multiplication of partial single amplified genomes (SAGs; Stepanauskas and Sieracki, 2007; Malmstrom et al., 2013; Kashtan et al., 2014; Berube et al., 2019; Nakayama et al., 2019) or metagenomes assembled genomes (MAGs; Iverson et al., 2012; Haro-Moreno et al., 2018). Due to their ubiquity, their natural abundance *in situ*, the occurrence of well-defined ecotypes and good knowledge of how environmental parameters influence their biogeography, marine picocyanobacteria constitute excellent model organisms to tackle evolutionary processes involved in niche partitioning.

*Synechococcus* and *Prochlorococcus* are the two most abundant photosynthetic organisms on Earth (Partensky et al., 1999a; Scanlan, 2012). As major primary producers, they have a pivotal role in CO_2_ fixation and carbon export and are key players in marine trophic networks (Jardillier et al., 2010; Flombaum et al., 2013; Guidi et al., 2016). Although these organisms often co-occur in (sub)tropical and temperate waters, *Synechococcus* is present from the equator to sub-polar waters, while the distribution of *Prochlorococcus* is restricted to the latitudinal band between 45°N and 40°S (Johnson et al., 2006; Flombaum et al., 2013; Paulsen et al., 2016). This broad distribution implies that these two microorganisms are able to survive in a large range of environmental niches along *in situ* gradients of temperature, light intensity as well as micro- and macro-nutrients (Bouman et al., 2006; Zwirglmaier et al., 2008; Scanlan, 2012; Sohm et al., 2015; Farrant et al., 2016).

The ability of marine picocyanobacteria to occupy various niches is likely related to the high intrinsic genetic diversity of these taxa. The *Synechococcus*/*Cyanobium* radiation has been split into three main groups, called Sub-Clusters (hereafter SC) 5.1 to 5.3 (Dufresne et al., 2008; Huang et al., 2012). While members of SC 5.2, currently encompassing strains assigned to both the *Synechococcus* and *Cyanobium* genera, are restricted to near coastal and estuarine areas, SC 5.1 and 5.3 are mainly marine, with SC 5.1 dominating in most oceanic waters and showing the highest genetic diversity currently comprising 18 distinct clades and 40 sub-clades so far described (Ahlgren and Rocap, 2012; Mazard et al., 2012). The *Prochlorococcus* genus forms a branch at the base of the *Synechococcus* SC 5.1 radiation and although it includes seven major lineages, usually referred to as ‘clades,’ the whole genus is actually equivalent to a single marine *Synechococcus* clade from a phylogenetic viewpoint (Huang et al., 2012; Biller et al., 2015; Farrant et al., 2016). Lineages thriving in the upper mixed layer, so-called High Light-adapted (HL) clades, are genetically distinct from those occupying the bottom of the euphotic zone, so-called Low Light-adapted (LL) clades. Furthermore, while members of HLI were shown to colonize subtropical and temperate waters, HLII to IV are adapted to higher temperatures (Johnson et al., 2006; Zinser et al., 2007; Martiny et al., 2009b), with HLII colonizing N-poor areas and HLIII and IV being restricted to iron(Fe)-limited environments (Rusch et al., 2010; West et al., 2011; Malmstrom et al., 2013). For *Synechococcus*, distribution and environmental preferences have only been well characterized for the five dominant clades in the field (clades I to IV and CRD1). Members of clades I and IV have been shown to be cold thermotypes that dominate in coastal, mixed and/or high latitude, nutrient-rich waters, while clades II and III are warm thermotypes, predominating in N-depleted areas and P-depleted regions, respectively (Zwirglmaier et al., 2008; Scanlan et al., 2009; Pittera et al., 2014; Sohm et al., 2015; Farrant et al., 2016). Finally, members of clade CRD1 were recently found to be dominant in large Fe-depleted areas of the world Ocean (Sohm et al., 2015; Farrant et al., 2016). Even though clades globally occupy distinct niches, it was also shown that distinct ecotypes within *Prochlorococcus* and *Synechococcus* clades can display specific distribution patterns (Mazard et al., 2012; Kashtan et al., 2014; Larkin et al., 2016), with for instance distinct genetic groups within clades II and CRD1 colonizing different thermal niches (Farrant et al., 2016).

Despite good knowledge of both their genetic diversity and environmental preferences, little is known about how environmental factors influence genome diversity and shape the community structure of marine picocyanobacteria, especially for *Synechococcus*. However, the development of high throughput sequencing techniques now allows such questions to be addressed. In particular, comparative genomics approaches applied to bacteria have revealed the high variability of microbial gene content, even for closely related strains sometimes displaying identical 16S rRNA sequences (Konstantinidis and Tiedje, 2005b). They notably led to the definition of (i) the core genome, the conserved part of the genome that encompasses genes shared by all strains, and (ii) the flexible genome, the content of which is much more variable and dependent on the local biotic and abiotic environment (Lan and Reeves, 2000; Cordero and Polz, 2014). In cyanobacteria, previous studies based on multiple genome comparisons have shown that these organisms still present a so-called ‘open pan-genome’ (Tettelin et al., 2005; Baumdicker et al., 2012; Simm et al., 2015). Indeed, each newly sequenced genome brings novel genes without diversity saturation, and this holds true for *Prochlorococcus* and *Synechococcus*, for which only 14 (Kettler et al., 2007; Biller et al., 2014) and 17 genomes, respectively (Dufresne et al., 2008; Baumdicker et al., 2012) have so far been compared. These studies thus highlight that the genomic diversity of natural populations is still mostly under-sampled, which strongly limits the interpretation of comparative genomic analyses. Here, we use a dataset of 81 non-redundant genomes of marine or halotolerant picocyanobacteria, of which 34 are newly sequenced complete *Synechococcus* genomes, to further assess the genomic diversity within these genera and how occupancy of new realized niches has impacted the evolution of these genomes. Analysis of this unprecedented genome dataset with original bioinformatic tools allowed us to estimate the relative contribution of gene gains/losses and sequence divergence on the diversification of marine picocyanobacteria and to highlight key processes involved in their adaptation to various environmental niches.

## Results

### Picocyanobacteria Exhibit a Wide Intra-Clade Genomic Diversity

**FIGURE 1:**
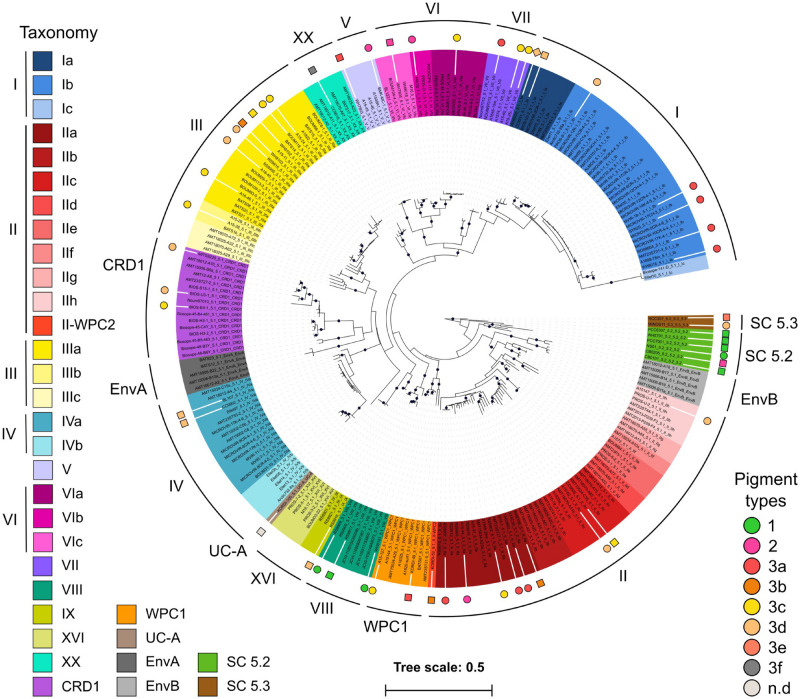
Phylogenetic position of the 53 mostly marine *Synechococcus* and *Cyanobium* genomes used in this study. A maximum-Likelihood tree was generated based on 231 *petB* sequences from both cultured and environmental samples. Black dots indicate bootstrap support over 70%. Sequences were named after strain name_sub-cluster_clade_subclade [sub-clade assignments as in Farrant et al. (2016)] and the background colors correspond to the finest possible taxonomic resolution obtained for each strain using the *petB* marker gene (left hand side legend). Colored circles surrounding the tree indicate newly sequenced genomes, while squares indicate previously available ones. Note that the WH8020 genome indicated by a diamond was not used in this study due to its poor quality. Symbols are colored according to their pigment type as defined previously (Humily et al., 2014; Xia et al., 2017b; Grébert et al., 2018; right hand side legend).

**FIGURE 2:**
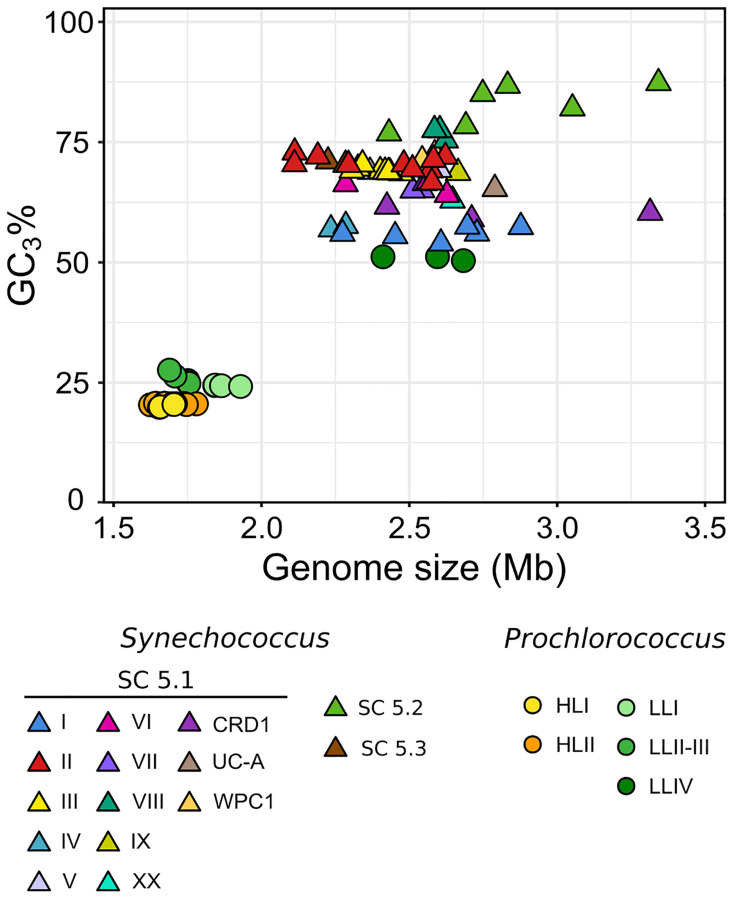
Relationship between genome size and GC3% (GC content at the third codon position). Each symbol corresponds to a different genome, with *Prochlorococcus* indicated by circles and *Synechococcus/Cyanobium* by triangles. The color of each symbol indicates the clade or SC.

In order to expand the coverage of *Synechococcus* in available marine picocyanobacterial genomes, 34 new strains were sequenced from cultured isolates, resulting in a quasi-doubling of the current number of complete or near-complete genomes publicly available for this genus. Strains were selected to cover the extent of the phylogenetic and pigment diversity of *Synechococcus*, as well as maximize their geographic origin and trophic regimes of their isolation site (Figure 1 and Supplementary Table S1). It should be noted though, that no cultured isolates are available yet for the EnvA and EnvB clades (Mazard et al., 2012; Farrant et al., 2016). The use of Wisescaffolder (Farrant et al., 2015) allowed us to close 28 out of the 31 genomes sequenced by the Genoscope and the Center for Genomic Research, with only one gap remaining in strains RS9915 and BOUM118 [both in the giant gene *swmB* (Brahamsha, 1996; McCarren and Brahamsha, 2007)] and three gaps in strain BIOS-E4-1 (two in genes encoding a PQQ enzyme repeat family protein and one in an LVIVD repeat family protein). This high-quality genome dataset constitutes a key asset for comparative genomics analyses. Consistent with the genome streamlining that occurred in most *Prochlorococcus* lineages (Dufresne et al., 2005, 2008; Kettler et al., 2007), average genome size and GC% are expectedly lower in *Prochlorococcus* (1.815 Mb and 34.8%, respectively) than in *Synechococcus*/*Cyanobium* (2.533 Mb and 59.18%, respectively), with genome sizes ranging from 1.625 Mb for *Prochlorococcus* HLII strain GP2 to 3.342 Mb for *Cyanobium gracile* PCC 6307 (SC 5.2) and GC% from 30.8% (EQPAC1, MED4, and MIT9515) to 68.7% (PCC 7001 and PCC 6307, Supplementary Table S1). Of note, members of the cold-adapted *Synechococcus* clades I and IV exhibited the lowest GC% values of all *Synechococcus*/*Cyanobium* strains (53.8 ± 0.73%) and this difference is even more marked using GC_3_%, i.e., the GC content at the third codon position (56.7 ± 1.25%; Figure 2; *p* < 10^–8^ Wilcoxon test for clades I and IV vs. all other *Synechococcus*/*Cyanobium*). By contrast, the warm-adapted clades II and III displayed significantly higher values (70.2 ± 1.5%; *p* < 10^–5^ Wilcoxon test clades II and III vs. clades I and IV), while the highest GC_3_% was found for members of the brackish strains of *Synechococcus* clade VIII and SC 5.2 (81.1 ± 4.6%; *p* < 10^–5^ Wilcoxon test clade VIII and SC 5.2 vs. all other *Synechococcus*). Thus, although the strongest GC_3_% variation was associated to genome reduction in *Prochlorococcus*, some of the GC_3_% variations might be related to the ecological niches occupied by these organisms and notably to thermal and variable salinity niches (Fuller et al., 2003).

**FIGURE 3:**
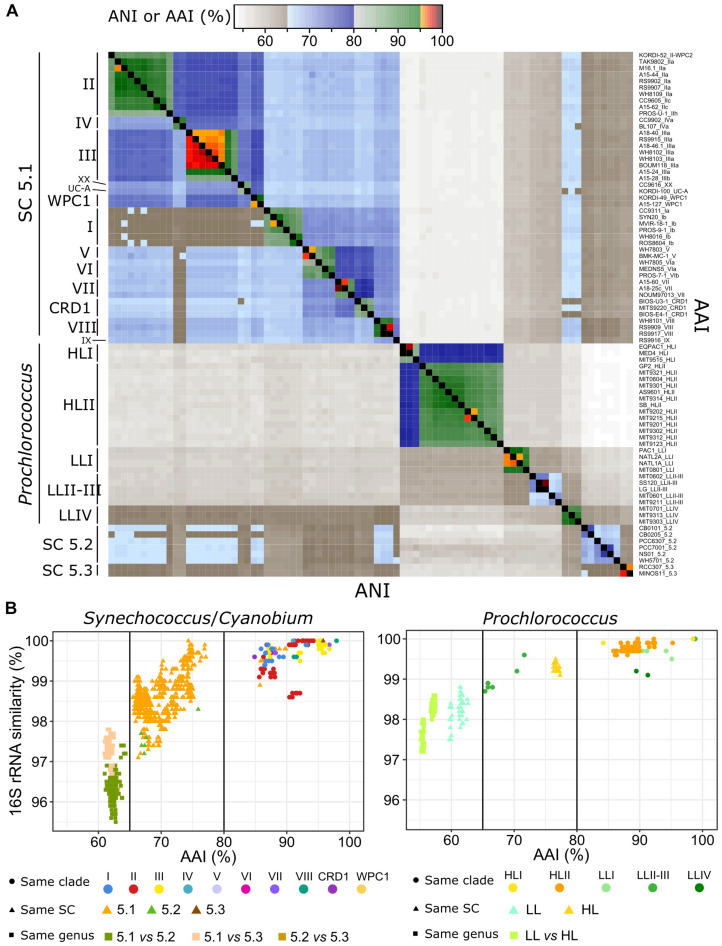
Genomic diversity of marine picocyanobacteria. **(A)** Heatmap of average nucleotide identity (ANI, bottom left triangle) and average amino acid identity (AAI, upper right triangle) between pairs of genomes. Each lane corresponds to a strain, and strains are ordered according to their phylogenetic relatedness. Strains are as labeled as strain_subclade (or higher taxonomic level when no sub-clade has been defined). **(B)** Relationships between 16S rRNA identity, AAI, and taxonomic information for *Synechococcus/Cyanobium* (left panel) and *Prochlorococcus* (right panel) genomes. Dots correspond to comparisons between pairs of genomes belonging to the same clade, triangles between pairs of genomes belonging to the same SC but different clades and squares between pairs of genomes belonging to different SC.

Although they all belong to a monophyletic, long diverged branch within the cyanobacteria radiation (Shih et al., 2013; Sánchez-Baracaldo, 2015), picocyanobacterial genomes show a tremendous diversity of both nucleotide sequences and gene content. Average nucleotide identity (ANI) and average amino acid identity (AAI) between pairs of picocyanobacterial genomes indeed ranged from 54.1 to 99.9% and 53.16 to 98.9%, respectively and intra-clade ANI and AAI were on average 91.8 and 91.04% (Figure 3A and Supplementary Figure S1). Thus, members of a given clade and even in most cases a given sub-clade, displayed ANI greater than 95%, classically used to define microbial species (Konstantinidis and Tiedje, 2005a; Goris et al., 2007). Interestingly, *Synechococcus* clades I and IV showed a particularly low ANI with other *Synechococcus* strains, while their ANIs with *Prochlorococcus* genomes were higher than for other *Synechococcus*-*Prochlorococcus* pairs. Since we did not observe this specificity with AAI, it is likely due to the low GC_3_% of *Synechococcus* clades I and IV (Figure 2).

A plot of the relationship between 16S rRNA identity and AAI for the different pairs of genomes (Figure 3B) additionally showed two major discontinuities. The first one at 80% AAI discriminated pairs of strains of the same clade from pairs of strains from different clades. Notable exceptions concerned the closely related and globally scarce *Synechococcus* clades V and VI as well as clades XX and UC-A, which fall within the intra-clade divergence level in terms of 16S rRNA identity and AAI, and *Prochlorococcus* clade LLII-III, which showed a divergence level similar to *Synechococcus* intra-SC divergence, suggesting that the gathering of these two clades into a single clade (Kettler et al., 2007; Biller et al., 2015) should be reconsidered, as suggested by Yan et al. (2018). The second discontinuity set apart *Synechococcus* strains of the same SC from strains of different SC (<98% 16S rRNA identity and <65% AAI), reflecting a very ancient genomic diversification between the three SC (see below). Because of this clear discontinuity, we propose to split *Synechocococcus* and *Cyanobium* into three distinct taxa: *Ca.* Marinosynechococcus (SC 5.1), *Cyanobium* (SC 5.2) and *Ca.* Juxtasynechococcus (SC 5.3). *Prochlorococcus* strains from different LL clades also fell below the 65% AAI discontinuity, highlighting the large divergence within this group. It is noteworthy that strains within SC 5.2 displayed a particularly low 16S rRNA identity compared to strains within SC 5.1, likely due to the low number of sequenced genomes relative to the wide diversity of this lineage, while in contrast the only two *Synechococcus* SC 5.3 genomes of our dataset were very closely related.

**FIGURE 4:**
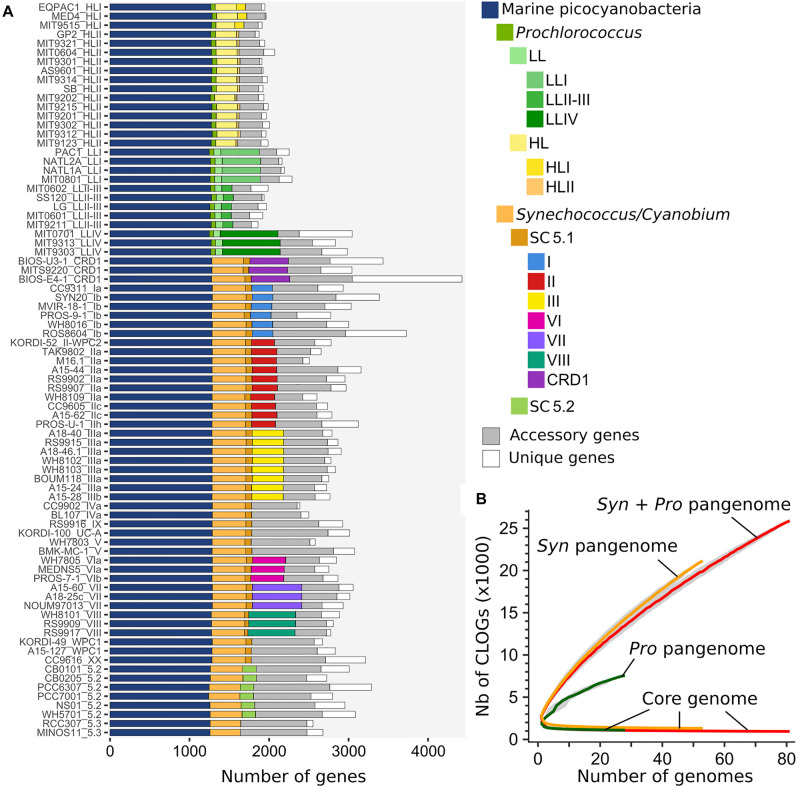
Core, accessory and pan genomes of marine picocyanobacteria. **(A)** Distribution of clusters of likely orthologous genes (CLOGs) in picocyanobacterial genomes. A CLOG is considered as core in a taxonomic group if it is present in ≥90% of the strains within this group. Sets of core CLOGS are inferred only for taxonomic groups with more than 3 genomes. Strains are labeled as strain_subclade (or higher taxonomic level when no sub-clade has been defined). **(B)** Evolution of the pan and core genomes for an increasing number of picocyanobacterial genomes (red, 81 genomes), *Synechococcus/Cyanobium* (orange, 53 genomes) and *Prochlorococcus* (green, 28 genomes). The gray zone around each curve represents the first and third quartiles around the median of 1,000 samplings by randomly modifying the order of genome integration.

In order to manually refine the annotation of these genomes and ease comparative genomic analyses in terms of gene content, all genomes were included in the Cyanorak v2.1 information system^1^, in which predicted genes were grouped into clusters of likely orthologous genes (CLOGs) by all-against-all sequence similarity. This clustering allowed us to determine the core genome, i.e., CLOGs present in all strains, and the pan-genome, i.e., all CLOGs present in at least one strain, at various phylogenetic depths (Tettelin et al., 2005). When considering the whole dataset, the number of core CLOGs as a function of the number of genomes showed an asymptotic decline, tending toward a core set of 911 genes (Figure 4B). In contrast, the pan-genome of marine picocyanobacteria, containing 27,376 CLOGs, was still far from saturation, revealing that even with 81 genomes, every newly sequenced picocyanobacterial genome still brought about 192 new genes. This result held true when considering *Prochlorococcus* (7,537 CLOGs) and *Synechococcus* (20,986 CLOGs) independently, indicating that we still missed an essential part of the genetic diversity within both genera that is yet to be sequenced from the field. A major asset brought by the 34 newly sequenced *Synechococcus* genomes is the availability of several genomes per clade, which allowed us to estimate the relative sizes of the core set of CLOGs at different taxonomic levels (i.e., genus, SC, and clades), the accessory genome, i.e., the non-core CLOGs shared at least by two strains, and, and unique genes, i.e., CLOGs present in a single strain (Figure 4A and Supplementary Table S2). While the proportion of accessory genes was pretty constant between genomes, constituting on average 13 ± 2.4% and 20.7 ± 6.3% of the *Prochlorococcus* and *Synechococcus* genomes, respectively, unique genes constituted the most variable part of the genomes, ranging from 0.6–21.9% and 1.5–31.2% of the *Prochlorococcus* and *Synechococcus* genomes, respectively, and were directly related to genome size. The newly sequenced strain BIOS-E4-1 (clade CRD1) contained by far the largest gene number of the genome dataset (4,426 genes), with a large proportion of unique genes (31.2%). Noteworthy, a significant proportion of CLOGs was present in all strains of a given clade (e.g., 335 genes for *Synechococcus* clade III, or 143 genes for *Prochlorococcus* HLI) and could thus potentially be involved in the adaptation of these taxa to specific environmental conditions. However, it should be noted that only a sub-set of these CLOGs were truly specific to each clade (e.g., 32 and 11 genes present in clades III and HLII, respectively; Supplementary Table S3) or ecologically significant taxonomic units (ESTU *sensu* (Farrant et al., 2016); see below and Supplementary Table S4), that is absent from all other clades or ESTUs.

### Dynamics of the Evolution of Gene Content in Marine Picocyanobacteria

**FIGURE 5:**
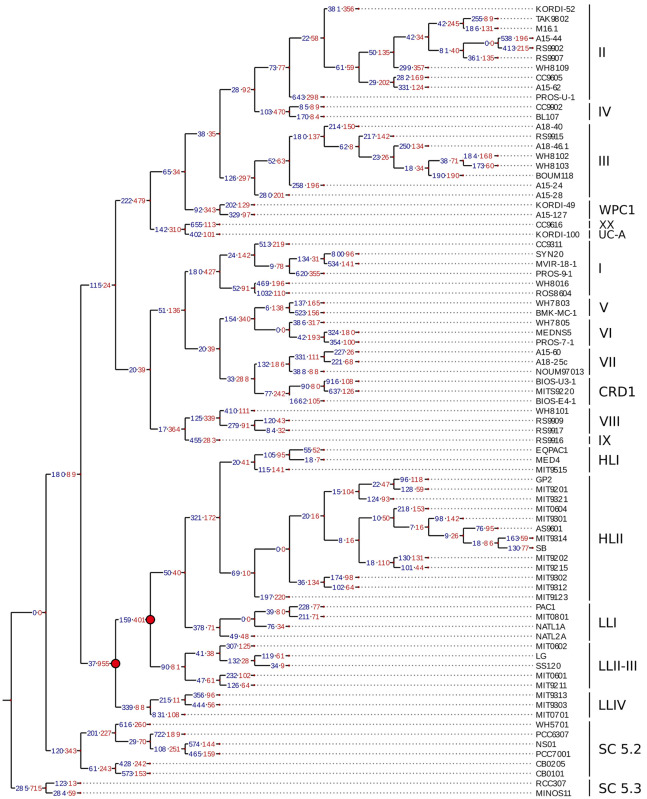
Estimation of the gene gains and losses during the evolution of marine picocyanobacteria. The ancestral state of presence/absence of every cluster of likely orthologous genes (CLOGs) was assessed using Count (Csurös, 2010) and used to infer the number of gains and losses of gene families on each branch of the tree using the phylogenetic core protein tree as reference. The number of gained and lost genes is labeled in blue and red, respectively. Nodes highlighted in red correspond to the major genome streamlining events that have occurred in the *Prochlorococcus* radiation.

To better understand the evolutionary processes that led to the diversification of gene content within marine picocyanobacterial genomes, we estimated by Maximum Likelihood the number of gene gain and loss events on each branch of a reference phylogenetic tree built from a concatenation of 821 single core proteins (Figure 5). As previously observed (Dufresne et al., 2005; Kettler et al., 2007), the gain and loss values obtained for *Prochlorococcus* were consistent with the scenario of a major genome streamlining process that occurred during the evolution of this genus, since an excess of gene loss was observed at the base of this radiation (Figure 5). Globally, the number of genes gained and lost on each branch of the picocyanobacterial tree was quite variable. While on internal branches the number of gains and losses remained limited and balanced for both *Prochlorococcus* and *Synechococcus* SC 5.1 (gains ≤ 378, losses ≤ 479; not taking into account the genome streamlining at the base of the *Prochlorococcus* radiation), a higher number of events were generally observed on terminal branches as well as an excess of gains compared to losses, with up to 1,662 gained genes on the branch leading to *Synechococcus* BIOS-E4-1 (SC 5.1) and 831 on the one leading to *Prochlorococcus* MIT0701, for 105 and 108 lost genes, respectively.

By using calibration time points from a previous study (Sánchez-Baracaldo, 2015), we estimated that this corresponds to about 0.71 and 4.62 genes gained (1.67 and 1.80 genes lost) per million years (My) on internal and terminal branches of *Synechococcus* SC 5.1, respectively, while internal and terminal branches of *Prochlorococcus* HL gained 1.45 and 4.5 genes (0.87 and 3.72 lost; Table 1). The higher values observed for the terminal branches are related to the high number of strain-specific genes and reflect the fact that most of the variability in gene content occurs at the ‘leaves’ of the tree. If we assume the rate of gene gain to be constant over time, this suggests that most of the genes gained on internal branches have been secondarily lost and are therefore not represented in our genomic dataset.

**TABLE 1:** Estimation of the number of gained, lost and/or fixed genes per million years (My) as well as total and fixed number of substitutions on internal branches (int. b.) or terminal branches (ter. b.) for *Prochlorococcus* (*Pro*) HL and *Synechococcus* (*Syn*) SC 5.1.

Rate (per My)		*Pro* HL	*Pro* HL	*Syn* SC 5.1	*Syn* SC 5.1
		int. b.	ter. b.	int. b.	ter. b.
Gene gain	Value	**1.45**	**4.5**	**0.72**	**4.62**
	*SE*	0.08	0.52	0.12	0.68
	Adj. *R*^2^	0.95	0.83	0.46	0.50
	*p*-value	<10^–5^	<10^–5^	<10^–5^	<10^–5^
Gene loss	Value	**0.87**	**3.72**	**1.68**	**1.8**
	*SE*	0.26	0.44	0.16	0.22
	Adj. *R*^2^	0.41	0.82	0.73	0.60
	*p*-value	4.7 × 10^–3^	<10^–5^	<10^–5^	<10^–5^
Specific gene fixation	Value	**0.39**	**–**	**0.16**	**–**
	*SE*	0.03	–	0.06	–
	Adj. *R*^2^	0.9	–	0.11	–
	*p*-value	<10^–5^	–	0.01	–
Amino acid substitutions	Value	**515.51**	**312.97**	**117.8**	**96.54**
	*SE*	17.2	9.11	3.64	1.86
	Adj. *R*^2^	0.98	0.99	0.96	0.98
	*p*-value	<10^–5^	<10^–5^	<10^–5^	<10^–5^
Specific amino acid fixation	Value	**78.1**	**–**	**18.41**	**–**
	*SE*	5.44	–	0.83	–
	Adj. *R*^2^	0.93	–	0.92	–
	*p*-value	<10^–5^	–	<10^–5^	–

*SE, standard error; adj. *R*^2^, adjusted *R*^2^.*

**FIGURE 6:**
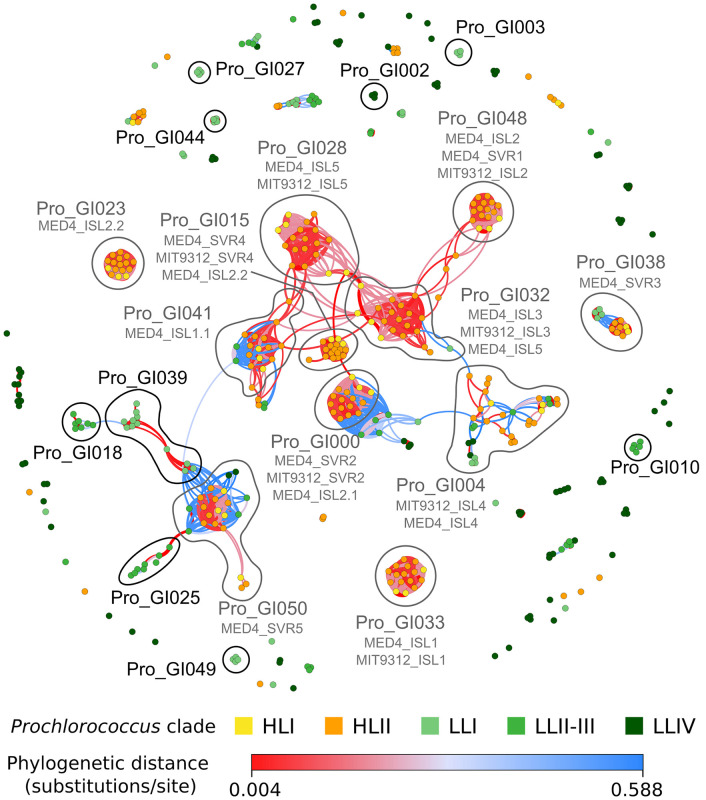
Network of shared gene islands between all *Prochlorococcus* strains analyzed in this study. Each node corresponds to a genomic island in a given strain, the gene content of which is listed in Supplementary Table S5. Edges were colored according to the phylogenetic distance between strains, with red indicating closely related strains and blue more distantly related strains, as indicated in the color bar. Edge width corresponds to the Jaccard distance between islands based on gene content. Nodes were colored based on *Prochlorococcus* clade. Modules cited in the text are surrounded with a gray line for those containing islands already described in the literature [subtitled with their names in Coleman et al. (2006) and Dufresne et al. (2008)] and a black line for new modules described in the present study. The gene and genomic island composition of each module is described in Supplementary Table S6.

As genomic islands have been shown to play a key role as repositories of laterally transferred genes potentially involved in niche adaptation in marine picocyanobacteria (Coleman et al., 2006; Kettler et al., 2007; Dufresne et al., 2008; Delmont and Eren, 2018; Yan et al., 2018), we explored the contents of these islands in all analyzed genomes. Most genomes were too distant to compare genomic islands between strains by whole genome alignment as performed by Coleman et al. (2006) on *Prochlorococcus*, so here genomic islands were defined in each strain as regions of the genome enriched in gained genes using a similar approach as Kettler et al. (2007) but with a threshold to define the limits of the islands in each strain (see section “Materials and Methods”; Supplementary Table S5). The number of gained genes located in genomic islands and shared by pairs of strains showed that closely related strains share many more island genes than distantly related ones and that only a few exchanges of genes occur between distantly related clades (Supplementary Figure S2). These observations are particularly striking for *Prochlorococcus* HL streamlined genomes that share only a low proportion of island genes with *Synechococcus.* A notable exception is *Synechococcus* clade VIII, which shares more island genes with strains of SC 5.2 than with most SC 5.1 strains, an expected pattern since these groups co-occur in coastal or estuarine waters of variable salinity (Fuller et al., 2003; Chen et al., 2006; Dufresne et al., 2008). To further explore how strains share genomic islands, we used an innovative network method based on the partial similarity of gene contents between islands shared by pairs of strains. It allowed us to retrieve islands previously identified either by direct pairwise comparison of *Prochlorococcus* HLI MED4 and HLII MIT9312 strains (Coleman et al., 2006) or by analyzing the deviation in tetranucleotide frequency in individual *Prochlorococcus* and *Synechococcus* genomes (Dufresne et al., 2008), demonstrating the validity of our automated approach (Supplementary Figure S3 and Supplementary Tables S6, S7). Interestingly, most islands identified by these authors in *Prochlorococcus* HL strains appeared to be shared by all HL strains, forming dense red, knot-shaped modules in the network (e.g., Pro_GI033 = MED4 ISL1; Pro_GI048 = MED ISL2; Pro_GI028 = MIT9312 ISL5; Pro_GI000 = MED SVR2; Pro_GI015 = MED4 SVR4; Pro_GI041 = MED4 ISL1.1; Pro_GI023 = MED4 ISL2.2; Figure 6 and Supplementary Table S6). These red knots correspond to genomic regions prone to gene integration that have likely been acquired by the common ancestor of all HL strains, then vertically transferred to all descendants, much like the phycobilisome region that is shared by all *Synechococcus* strains (Dufresne et al., 2008). In contrast, ISL4 island, initially identified in MED4 by Coleman et al. (2006) and later confirmed both by Dufresne et al. (2008) and our automated island detection approach (Pro_GI004; Supplementary Figure S3), does not form a red knot but only a fuzzy network of interconnected islands, each shared by only 2 to 4 strains (Figure 6). So this island, whose gene content is highly variable, has seemingly been more recently acquired by a subset of the HL population. Our approach also unveiled previously undescribed islands specifically shared by sets of *Prochlorococcus* LL strains, including Pro_GI027, 039, 044 and 049 specific to LLI strains [several being enriched in *hli* genes, known to be amplified in LLI compared to other LL strains; (Partensky and Garczarek, 2010)], Pro_GI010, 018 and 025 specific to LLII/III strains, and Pro_GI002 as well as 13 other modules specific to LLIV strains, including several containing genes encoding lanthipeptides (Tang and van der Donk, 2012; Figure 6 and Supplementary Table S6).

**FIGURE 7:**
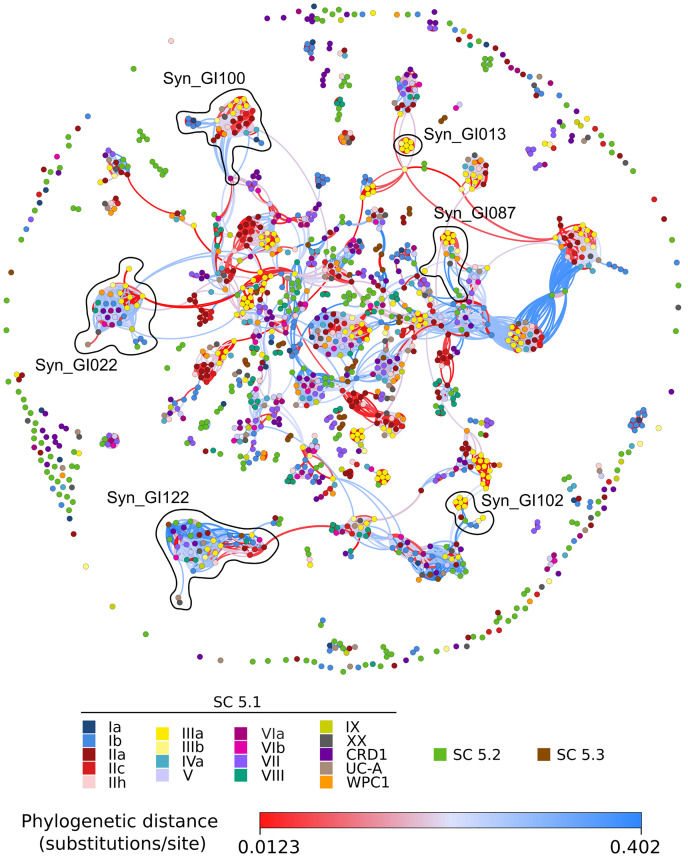
Same as Figure 6 but for marine *Synechococcus/Cyanobium* strains. The gene and genomic island composition of each module is described in Supplementary Table S7.

In *Synechococcus*, the network included relatively few dense red knots compared to *Prochlorococcus* (Figure 7). Among the most notable ones are three clade III-specific islands: the first one (Syn_GI013) gathers a gene cluster (*cynA-B-D*) involved in cyanate transport (Kamennaya and Post, 2011; Supplementary Table S7); the second one (Syn_GI087) includes a specific beta-glycosyltransferase and *swmA*, a protein involved in a special type of motility observed only in members of this clade (McCarren et al., 2005); the third one (Syn_GI102) notably contains *swmB*, encoding a giant protein also involved in this motility process (McCarren and Brahamsha, 2007). Another interesting example is Syn_GI100, which notably encompasses a 3-gene cluster composed of one *nfeD* homolog and two flotillin-like genes that both have similarity to the *floT* gene involved in the production of lipid rafts, whose deletion in *Bacillus subtilis* was found to strongly affect cell shape and motility (Dempwolff et al., 2012). Interestingly, this gene cluster was found in the only two clade III strains (A15-24 and A15-28) that lack *swmA* and *swmB* as well as in several distantly related strains. Conversely, no *swmA-B*-containing strain was found to possess the *nfeD-floT1-floT2* gene cluster. The network approach also detected quite a few knots containing both red and blue edges. The latter color indicates that strains sharing these islands are distantly related to one another. Thus, knots that are mixing red and blue edges potentially emphasize relatively recent horizontal gene transfers between clades or longer phylogenetic distances. This includes (i) Syn_GI022, a module found in many SC 5.1 strains with the notable exception of clade II strains, which encompasses a large gene cluster involved in glycine betaine synthesis (*gbmt1-*2) and transport (*proV-W-X*), located in some strains next to another gene cluster involved in the biosynthesis of glucosylglycerate [*gpgS-gmgG-gpgG*; (Scanlan et al., 2009)] and (ii) Syn_GI122, a module comprising strains from almost all lineages that encompasses genes encoding uncharacterized cell surface proteins, secreted CHAT domain-containing proteins and/or genes involved in the biosynthesis of cyclic AMP (cAMP), including adenylate cyclases located in the vicinity of cyclic nucleotide-binding proteins, such as the cAMP receptor protein (CRP) or a cAMP-regulated small-conductance mechanosensitive ion channel. Altogether, this network approach nicely complements the detection of genomic islands in single genomes by providing insights about the evolutionary history of these genomic islands.

### Relative Contributions of Variability at the Sequence and Gene Content Levels in the Evolution of Picocyanobacteria

**FIGURE 8:**
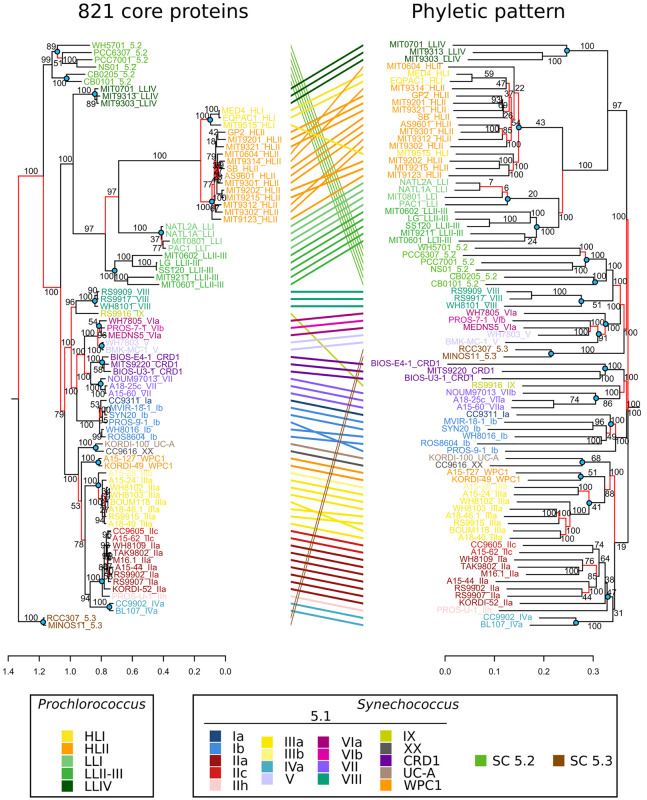
Comparison of phylogenies based on core protein sequences and phyletic patterns of non-core genes. *Left*, Maximum Likelihood tree based on the alignment of 821 concatenated core proteins. *Right*, Neighbor-Joining tree based on the Jaccard distance between the phyletic patterns of 27,376 accessory gene families found in the 81 picocyanobacterial genomes. Labels are colored according to the strain sub-clade. Red branches indicate discrepancies between the topology of the two trees. Nodes located at the base of a clade and highlighted by blue dots were used for branch length comparisons in Supplementary Figure S4.

The fairly low rate of gene acquisition evidenced in this study raises the question of the relative weight of gene content variations vs. substitutions in the nucleotide sequence in the long-term diversification and adaptation processes of these organisms. Figure 8 compares a phylogenetic tree built with a concatenation of 821 picocyanobacterial core protein sequences to a dendrogram based on the phyletic pattern (i.e., the pattern of presence/absence of each CLOG in each strain). Topologies of the two trees were globally similar, which reveals that fixation of genes and fixation of mutations occurred concomitantly during the evolutionary history of marine picocyanobacteria. Yet, *Synechococcus* clade VIII and SC 5.2 were found to be closely related in the dendrogram based on the phyletic pattern. Indeed, as previously reported in a study using 11 *Synechococcus* genomes (Dufresne et al., 2008), these taxa share a fair number of genes, potentially related to their co-occurrence in brackish environments. Interestingly, the closely related clades V and VI cluster together with these two taxa, indicating that they may also share with clade VIII and SC 5.2 some mechanisms of adaptation to low salinity niches (see below). Although the presence of SC 5.3 has been recently documented in freshwater environments (Cabello-Yeves et al., 2017), the presence of the two marine sequenced strains (RCC307 and MINOS11) at the base of this halotolerant group might instead be due to attraction by SC 5.2.

Among the *Synechococcus* SC 5.1 and *Prochlorococcus* radiations, we identified a few incongruences between the two trees within *Synechococcus* clades I, II, III, and VI and *Prochlorococcus* HLII (Figure 8) that are likely due to the relatively low number of specific genes within these clades. It is also worth noting that some clades were closer in terms of gene content than expected from the core phylogeny, in particular *Synechococcus* clades WPC1, XX and UC-A grouping with clade III in the tree based on the phyletic pattern. Finally, some clades lost their monophyly in the tree based on phyletic pattern, such as *Synechococcus* clades V and VI that were mixed together or *Prochlorococcus* HLI that was found to be mixed with HLII. This example is particularly interesting, since despite their clearly distinct phylogenetic clustering based on protein sequences and well-known ecological and physiological differences (Johnson et al., 2006; Martiny et al., 2009b), these two clades have a quite similar gene content, with only a few genes (29) present in all HLII strains but not in all HL strains (Figure 4A). Similarly, *Prochlorococcus* clade LLI, which was previously shown to occupy an intermediate niche between HL and strict LL members (LLII-IV) and to share genes with both ecotypes (Johnson et al., 2006; Partensky and Garczarek, 2010), actually appeared to share more genes with the LLII-III clade (1,382 genes) than with HL (1,290 genes). Altogether, these two examples show that within *Prochlorococcus*, although HL and LL have different gene contents, differentiation within HL and to a lesser extent within LLI-III rather relies on substitution accumulations than on variation in gene content.

Another major difference between these trees concerned branch lengths. By computing for each node at the base of a clade (blue dots in Figure 8) the average length from the node to its descending leaves (terminal length), and the length from the node to its parent node (internal length), we showed that the ratio of terminal to internal branch lengths was significantly higher (Mann–Whitney paired test, *p*-value < 0.0015) in the phyletic pattern tree than in the core tree (Supplementary Figure S4). This suggests that there were more amino acid substitutions before the divergence of clades than after, whereas there was more gene content variation between strains of a clade than between clades. In other words, this comparison revealed that most of the changes that were fixed in the long term by evolution are substitutions and not changes in gene content.

In order to quantify more precisely this difference, we compared the estimated number of gene gains and losses per My (Supplementary Figure S5) to the number of amino acid substitutions in core proteins per My (Supplementary Figure S6) and results of these comparisons are shown in Table 1. It is important to note that the rates of gene gain/loss and amino acid substitutions calculated this way should only be considered as lower bound estimates for several reasons. First, since we only have access to the present-day genomes and not to ancestral ones, measurements of the rate of genes gained in fact refer to genes gained and successfully retained over time in at least one strain. Second, the amino acid substitution rates were measured on core proteins, whose genes likely undergo a strong purifying selection. This, together with the much longer generation time of picocyanobacteria compared to model bacteria and with their considerable population size (Partensky et al., 1999b; Dufresne et al., 2005; Flombaum et al., 2013), could explain why estimated rates were lower than for other bacterial lineages (Lawrence and Ochman, 1998; McDonald and Currie, 2017). With this caveat in mind, in *Prochlorococcus* HL, 356× more amino acid substitutions than gene gains were estimated for internal branches per My, and 69.6× for terminal branches, primarily due to a higher rate of gene gain in the latter branches. In *Synechococcus* SC 5.1, a ratio of 164 and 20 was obtained for internal and terminal branches, respectively, the difference between the two genera likely being due to the higher rate of protein sequence evolution observed in *Prochlorococcus* (Dufresne et al., 2005).

We also compared at each node the fixation rate of amino acid substitutions in core proteins (i.e., amino acids in the alignment that are identical in all descending strains and different in all other strains) to the fixation rate of genes (i.e., present in all descending strains and in no other strain). 201× more amino acid variants than genes were fixed per My in *Prochlorococcus* HL (and 116× more for *Synechococcus* SC 5.1). This corresponds to a fixation rate of 78 and 18 amino acid changes in core proteins per My for *Prochlorococcus* HL and *Synechococcus* SC 5.1, respectively, while one gene is fixed once every 2.6 My for *Prochlorococcus* HL and once every 6.3 My for *Synechococcus* SC 5.1. While these numbers show that substitutions played a major role in genomic diversification, the question remains as what part of this diversification is related to an adaptive process.

### Role of Gene Content in the Adaptation of *Synechococcus* to Specific Niches

In contrast to *Prochlorococcus* (Kettler et al., 2007; Partensky and Garczarek, 2010; Biller et al., 2015; Delmont and Eren, 2018; Yan et al., 2018), few genomic diversity studies have been conducted so far in *Synechococcus*. In order to reveal whether the presence or absence of genes might be related to *Synechococcus* adaptation to specific niches, we defined sets of clades co-occurring in the field and occupying similar niches, based on assemblages of ESTUs as defined in Farrant et al. (2016). We then searched for genes occurring in strains within a given set and absent from other picocyanobacterial strains using a relaxed, niche-related definition of specificity (Supplementary Table S4). These analyses revealed only 18 CLOGs specific to members of both cold thermotypes, clades I and IV, among which 6 had a putative function, though with seemingly no direct relationship with adaptation to low temperature. However, the set of 19 CLOGs specific to clade I includes a particular isoform of the chaperone protein DnaK (DnaK4, CK_00056929; Supplementary Table S3) in addition to the three gene copies present in most *Synechococcus* SC 5.1 strains. This additional copy might be involved in protein folding in cold conditions (Genevaux et al., 2007).

Members of clades III, WPC1 and SC 5.3, co-occurring in warm, P-depleted oligotrophic waters, were found to share a much higher number of genes (85; Supplementary Table S4), among which 2 were previously reported to be related to phosphate availability: a yet uncharacterized gene (CK_00002088) found to be downregulated in early phosphate stress (Tetu et al., 2009) and a chromate transporter (ChrA), which was recently suggested to be involved in phosphate acquisition in *Prochlorococcus*, based on its enrichment in P-poor oligotrophic areas (Kent et al., 2016). Clades III and WPC1 also share a cluster of 12 consecutive genes potentially involved in capsular polysaccharide synthesis and export (including genes similar to *kps* genes in *Escherichia coli* K1, responsible for the formation of a polysialic acid extracellular capsule; see Kps cluster in Supplementary Table S4) and another cluster of 7 genes that might be involved in the use of organic nitrogen sources since it encompasses a putative nitrilase (CK_00002256). Additionally, 32 genes were found to be specific to the 8 clade III strains, including the above-mentioned cyanate transporter genes (*cynABD;* Kamennaya and Post, 2011) as well as a phosphate starvation-induced protein (PsiP1; Scanlan and West, 2002) and a specific alkaline phosphatase (CK_00052500) that potentially hydrolyses extracellular organic phosphates (Supplementary Table S3). Similarly, the two members of SC 5.3 also share a large number of strictly specific genes (215), including a regulator of phosphate uptake (PhoU; CK_00005756; diCenzo et al., 2017) as well as two putative phosphatases (CK_00005504, CK_00005619) and a putative pyrophosphatase (CK_00005811), in addition to the 4 putative pyrophosphatases present in most picocyanobacterial genomes (CK_00000642, CK_00000654, CK_00000805, and CK_00008108; Supplementary Table S3). Altogether, these results suggest that the occurrence of these genes might contribute to the success of clade III, WPC1 and SC 5.3 cells in oligotrophic, P-depleted environments such as the Mediterranean Sea in summer (Farrant et al., 2016), and indicates that members of these three taxa have adopted partially different strategies to cope with P depletion. To further explore the adaptive strategies of these clades to cope with low inorganic P concentrations, we compiled a Table displaying the number of copies of each CLOG related to P transport and metabolism in all *Synechococcus* strains (Supplementary Table S8). All clade III strains share at least three copies encoding the PstS transporter and one copy of *sphX*, in addition to the abovementioned ChrA transporter. The number of transporters is also high in clades VIII, WPC1 and members of SC5.2, while it is systematically lower in clades I, II, IV, VII, and CRD1. Interestingly, clades I, II, and IV strains virtually all lack *sphX*, with only one clade II strain (A15–44) possessing this gene. All members of clades I and IV also lack the genes *phoB* and *phoR* coding for the two-component system involved in P sensing and the regulation of P metabolism, as previously observed on fewer strains (Scanlan et al., 2009). While all clades have the genetic potential for phosphonate utilization, only some clade II strains and a single strain from clade III (A15–28) possess the genes for phosphite assimilation. This trait is, however, not conserved at the clade level. Finally, this detailed analysis revealed the particularly high number of shared phosphatase genes in clades III (8–12 genes) and WPC1 (8 and 11 genes, median = 9), in contrast to the lower number observed in clades I, II, IV, and VII (3–6, median = 5). This suggests an adaptive strategy to diversify sources of organic phosphate available to members of these clades, likely as an adaptation to environments depleted in inorganic P. Clade VIII seems to have specialized in a specific organic source with 3 or 4 copies of the same phosphatase while clades V, VI, CRD1 and SC5.2 have more variable numbers of phosphatases, reflecting strain-level variation rather than clade-level strategies.

Genes potentially involved in niche adaptation were also found in all three strains of the CRD1 clade, known to dominate in iron-depleted oceanic regions, which share a quite high number of specific CLOGs (81, Supplementary Tables S3, S4), though most of them have no known function. Among the characterized ones were a second copy of the flavodoxin IsiB, a Cu-containing protein known to replace ferredoxin in iron-depleted conditions (Erdner and Anderson, 1999), the ferrous iron transport protein FeoA, an iron-sulfur cluster biosynthesis family protein (CK_00008433) as well as 3 specific high light-induced proteins (HLIPs) that might provide protection from oxidative stress to photosystems (He et al., 2001).

Finally, in agreement with their clustering in the dendrogram based on phyletic pattern (Figure 8), clades VIII and SC 5.2 share 28 genes including a few strictly specific genes (Supplementary Table S4), such as a fatty acid hydroxylase (CK_00002851) involved in lipid biosynthesis, and one or two copies of a P-type ATPase (CK_00045881), a family of ATP-driven pumps known to transport a variety of different ions and phospholipids across membranes (Axelsen and Palmgren, 1998). It is also noteworthy that SC 5.2 and clade VIII share a fair number of genes potentially involved in the adaptation to low salinity with members of clades V, VI and sometimes VII, whose ecological niches are still poorly known (Zwirglmaier et al., 2008; Farrant et al., 2016; Xia et al., 2017a) and possibly encompass environments with variable salinity (Supplementary Table S4). This includes a specific small-conductance mechanosensitive ion channel (MscS family) that might be involved in the response to osmotic stress (CK_00056919; Haswell et al., 2011) and a bacterial regulatory protein of the ArsR family that besides regulating the efflux of arsenic and arsenite was suggested to participate in salt tolerance in *Staphylococcus aureus* through a Na^+^ efflux activity (Scybert et al., 2003). In addition, members of clade VIII share 22 specific genes, including a second potential mechanosensitive ion channel (MscS*;* CK_00056915), while members of SC 5.2 share 31 specific genes, including another *mscS* gene copy (CK_00003081) as well as genes encoding a putative chloride channel (CK_00042275) and a NAD-dependent malic enzyme, a protein known to be enhanced under salt stress in plants (Liu et al., 2007; Supplementary Table S3). Despite these few examples, it seems that the number of genes potentially related to the ecological niche occupied by each clade or assemblage of clades is fairly limited and varies depending on the considered niche, with for instance few genes related to thermal niche adaptation. Most of the diversity in gene content therefore relies on differences between individual strains rather than between phylogenetic groups or ESTUs, a large proportion of the sparsely distributed genes having yet unknown functions, some potentially being involved in niche adaptation.

### Role of Substitutions in Adaptation

Given our observation that a high number of amino acid substitutions have been fixed in the long term, we also searched for those potentially involved in niche adaptation. We identified “specific variants” as positions in core protein alignments for which a particular amino acid is found in all strains of a given clade, ESTU or set of ESTUs and a different amino acid is found in other strains. In order to reduce the noise due to the accumulation of clade-specific substitutions and to better identify the niche adaptation signal, we focused on variants shared by clades I and IV, which do not form a monophyletic group (Figure 8, left) but usually co-occur in cold, temperate waters (Zwirglmaier et al., 2007, 2008; Martiny et al., 2009b; Sohm et al., 2015; Farrant et al., 2016; Kent et al., 2019). We identified 180 proteins mainly involved in (i) energy metabolism, (ii) biosynthesis of cofactors, prosthetic groups and carriers, such as pigments and vitamins, (iii) protein synthesis and protein fate, and to a lesser extent (iv) transport and DNA metabolism (Supplementary Table S9). The first category encompassed proteins responsible for carbon fixation (the RuBisCO subunits RbcS and RbcL, the carbonic anhydrase CsoSCA, the carboxysome proteins CsoS1E and CsoS2, and the Calvin cycle enzyme Fbp-Sbp), two photosystem II subunits (the extrinsic PsbU protein and the manganese cluster assembly protein, Psb27) and a number of proteins involved in electron transport for photosynthesis and/or respiration (CtcAI, CtcEI, NdhA and two ATP synthase subunits: AtpA and AtpD). Furthermore, this set includes six proteins potentially involved in the response to light or oxidative stress: two High Light Inducible Proteins (HLIPs; CK_00001609 and CK_00001414), two peroxiredoxins (PrxQ), a glutaredoxin (CK_00000445) and a flavoprotein involved in the Mehler reaction (Flv1). We also identified a few enzymes involved in sugar metabolism and in particular in the pentose phosphate pathway (Pgl, TalA, and Zwf). As concerns the ‘protein synthesis’ and ‘protein fate’ categories, this includes six ribosomal proteins and nine amino acid biosynthesis proteins, several tRNA/rRNA modification enzymes and tRNA aminoacyltransferases as well as seven proteins responsible for folding and stabilization of polypeptides. Of particular interest are the proteins belonging to the ‘biosynthesis of cofactors, prosthetic groups and carriers’ category, including enzymes involved in chlorophyll (HemC, ChlN, and ChlB), cobalamin (CobO, cobQ, and CobU-CobP) and carotenoid biosynthesis. The latter includes CrtE and GpcE, two enzymes involved in the phytoene biosynthesis pathway and CrtP, CrtQ, and CrtL-b, the three enzymes catalyzing all the steps required to transform phytoene into β-carotene. It is also interesting to note that the five proteins displaying the largest number of specific substitutions relative to protein length are a putative ABC multidrug efflux transporter (CK_00008042; 19 positions specific to clades I and IV out of 607 amino acids), lycopene β-cyclase, responsible for the last step of β-carotene synthesis (CrtL-b; 7/347), the bifunctional enzyme fructose-1,6-biphosphatase/sedoheptulose-1,7-biphosphate phosphatase involved in both Calvin cycle and glycolysis (7/347), the photosystem II manganese cluster assembly protein Psb27 (3/160) and the ribosomal protein RpmB (1/78). Even though the number of substitutions is not directly correlated to the level of selection pressure, the high proportion of specific substitutions in these proteins suggests that they have been subjected to positive selection and therefore have potentially a role in adaptation to cold environments.

## Discussion

The availability of 81 complete and closed picocyanobacterial genomes with extensive manually refined annotations, including 34 novel *Synechococcus*, constitutes a key asset for comparative genomics analyses. With regard to previous studies (see e.g., Kettler et al., 2007; Dufresne et al., 2008; Scanlan et al., 2009), sequencing of several strains for most major *Synechococcus* clades revealed that the extent of genomic diversity is tremendous, at all taxonomic levels including within clades and most sub-clades. As previously observed for SAR11 (Nayfach et al., 2016; Tsementzi et al., 2016), ANI and AAI were indeed most often well below the cut-off of 95% (Figure 3), usually considered to be the limit between bacterial species (Konstantinidis and Tiedje, 2005a,b; Jain et al., 2018). Thus, based on this cut-off, most clades within cluster 5 *sensu* (Herdman et al., 2001) would correspond to one or even several species, as suggested by one research group (Thompson et al., 2013; Coutinho et al., 2016a,b). However, the delineation of so many species in a radiation that mostly exhibits a continuum in terms of within clade sequence identity (ID% range: 84–100%; Figure 3B) would create more confusion than clarification as it would result in most cases into single-strain species, which cannot be clearly differentiated based on their fundamental (see e.g., Moore and Chisholm, 1999; Pittera et al., 2014) and/or environmental realized niches (Huang et al., 2012; Sohm et al., 2015; Farrant et al., 2016; Kent et al., 2016). With this caveat in mind, it is clear that besides the *Prochlorococcus* lineage, there are three extremely divergent monophyletic groups within the marine *Synechococcus*/*Cyanobium* radiation (Sánchez-Baracaldo et al., 2019), which furthermore can be clearly discriminated based on 16S similarity vs. AAI plots (Figure 3B), with an AAI divergence below the 65% limit that has been proposed to discriminate distinct genera (Konstantinidis and Tiedje, 2007). Based on these criteria, our proposition to split the marine *Synechococcus* group into three distinct taxa: *Ca.* Marinosynechococcus (SC 5.1), *Cyanobium* (SC 5.2), and *Ca.* Juxtasynechococcus (SC 5.3). This proposal notably solves the inconsistency to have a mix of strains named *Cyanobium* spp. and *Synechococcus* spp. within SC 5.2, which should clearly all be called *Cyanobium* spp. For the universal acceptance of the revised taxonomy of this group and cyanobacteria at large (Komárek, 2016), both temporary names proposed for SC 5.1 and 5.3 as well as the potential definition of species within each of these radiations await validation by a large panel of cyanobacterial community members. In any case, any creation of new species within this group should likely take into account previously defined monophyletic clades and subclades as these phylogenetic groups have been used in most previous laboratory and environmental studies, whatever the genetic marker used (Palenik et al., 1997; Penno et al., 2006; Ahlgren and Rocap, 2012; Huang et al., 2012; Mazard et al., 2012; Scanlan, 2012).

The particularly high degree of genomic divergence occurring within Cyanobacteria Cluster 5 needs to be taken into account when putting results from comparative genomics of marine picocyanobacteria in the context of other highly sequenced bacterial groups such as pathogens and commensals (Harris et al., 2010; Kennemann et al., 2011; Mather et al., 2013). While high divergence and associated low level of synteny somehow limit the application of classical population genetics approaches, such as calculation of recombination rates (McDonald and Currie, 2017), our dataset is in contrast well suited to study the long-term evolutionary processes that have shaped the genomes of these abundant and widespread organisms in relation to their ecological niche occupancy. Comparative genomic analyses on marine picocyanobacteria have so far mainly focused on comparing gene repertoires from strains isolated from distinct niches, with the idea that niche adaptation largely relies on differential gene content (Rocap et al., 2003; Palenik et al., 2006; Kettler et al., 2007; Dufresne et al., 2008). Here, a comparison of several strains per clade led in most cases to the identification of relatively few specific genes of known function that may confer a trait necessary for niche adaptation, even using relaxed stringency criteria (e.g., by selecting genes present in >80 or 90% of strains within a clade/ESTU assemblage and in <20 or 10% of others; Supplementary Tables S3, S4). This may be due to the existence of an extended within-taxa microdiversity (Martiny et al., 2009b; Kashtan et al., 2014; Farrant et al., 2016; Larkin et al., 2016), where the more genomes in a taxon, the lower the number of genes found in all strains of this taxon. This fairly low number of niche-specific genes might also suggest that gene gain/loss, and fixation of these events during evolution, is a less prominent process to explain niche adaptation of marine picocyanobacteria than previously thought. Although lateral gene transfer is often considered to “commonly” occur between cells, and was notably shown to be involved in adaptation to nitrogen- or phosphorus-poor conditions in *Prochlorococcus*, no previous study explicitly stated the evolutionary time scale at which these adaptations took place (Martiny et al., 2006, 2009a; Kettler et al., 2007; Dufresne et al., 2008; Scanlan et al., 2009; Berube et al., 2014; Yan et al., 2018). Here, although the higher estimated rate of gene gains on the terminal branches of the phylogenetic tree indicates that most detectable events occurred fairly recently with regard to the long evolutionary history of both genera (Figure 5 and Table 1), adding time calibration to the tree led to an estimation of only 4.5 and 5.6 genes gained per My on terminal branches in *Prochlorococcus* HL and *Synechococcus* SC 5.1 strains, respectively. Thus, gene gains appear to be rather rare events. Even though these rates are approximate due to uncertainties in time calibration and probably underestimated, they are entirely in line with those estimated for *Prochlorococcus* HLII populations, thought to have diverged a few million years ago but only possessing a dozen unique genes (Kashtan et al., 2014). Furthermore, in accordance with previous studies on other bacterial groups (Lerat et al., 2005; Ochman et al., 2005; Nowell et al., 2014; McDonald and Currie, 2017), the fact that rates of gene gain/loss are estimated to be higher on terminal branches of the tree (Supplementary Figure S4), together with the high number of unique genes in every sequenced strain (Figure 4A), clearly suggests that most recently acquired genes will not be kept in the long term in both genera. Our calculation indeed gives an approximate value of 1.45 and 0.71 genes gained and subsequently kept per My in *Prochlorococcus* HL and *Synechococcus* SC 5.1, respectively (Table 1). This low fixation rate suggests that most of the recently gained genes have no or little beneficial effect on fitness in the long term and that we observe them in genomes because purging selection has not deleted them yet (Hao and Golding, 2006; Abby and Daubin, 2007; Rocha, 2008). Still, these recently gained genes could be involved in more transient adaptation processes at the evolutionary scale such as biotic interactions (e.g., resistance to viral attacks or grazing pressure).

Such a result also has important implications for interpreting the role of flexible genomes in the context of adaptation to distinct niches. Indeed, genes conferring adaptation to a specific niche are mixed in the genomes with genes with no or little beneficial effect and are thus difficult to identify – in particular when they have only a putative function. The relatively low gene fixation rate that we observed (Table 1) also implies that flexible genes that are fixed within a clade (i.e., clade-specific genes) were gained tens of millions of years ago, and thus might be more reflective of past selective forces than of recent adaptation to newly colonized niches. In this context, genes specifically shared by *Synechococcus* clade VIII and SC 5.2 suggest that adaptation to low salinity environments was a critical factor in their differentiation from other taxa and the most parsimonious evolutionary scenario would be a lateral transfer of these genes from a SC5.2-like strain to the common ancestor of clade VIII, which might date back to 51.6 My (confidence interval 0–141 My). Similarly, adaptation to phosphorus-depleted oligotrophic areas might have driven the differentiation of *Synechococcus* clade III, as revealed by the occurrence of P- and other nutrient-uptake genes specific to this clade. Interestingly, co-occurring ESTUs IIIA, WPC1A, and SC 5.3A only share a few common genes potentially involved in the adaptation to this limitation. Instead, these ESTUs seem to have independently acquired different sets of genes to improve P-uptake and/or assimilation and potentially use different sources of organic phosphate (see section “Results” and Supplementary Tables S4, S8). It is notable that some clade II strains have also potentially adapted to inorganic P depletion by acquiring or conserving the ability to use phosphite. It is also noteworthy in this context that in *Prochlorococcus*, P metabolism is not clade-related but dependent on within-clade variability in the gene content of specific genomic islands (Martiny et al., 2006, 2009a), further highlighting the variety of evolutionary paths that led to adaptation to low-P environments in these different lineages.

As proposed recently for other bacterial model organisms (Thrash et al., 2014; McDonald and Currie, 2017), natural selection of specific substitutions also appears to play a crucial role in genome diversification of marine picocyanobacteria and to have driven their adaptation to specific environments. Indeed, in the time necessary for one gene to be gained, we found that 20–60 amino acid substitutions accumulate in any picocyanobacterial genome (as estimated based on terminal branches of the phylogeny, Table 1). This finding brings new evidence to support the “Maestro Microbe” model of bacterial genome evolution recently proposed by Larkin and Martiny (2017), which posits that some phenotypic traits, such as thermal preferences, evolve by progressive fitness optimization of protein sequences rather than gene gains and losses. This theory is mainly based on the lack of specific genes that may explain trait differences between closely related organisms inhabiting distinct niches, and one of the best examples concerns *Prochlorococcus* clades colonizing temperate (HLI) and warm (HLII) environments (Coleman et al., 2006; Martiny et al., 2006; Kettler et al., 2007; Larkin and Martiny, 2017), which were partly mixed on our tree based on gene content despite a clear phylogenetic separation based on core marker genes (Figure 8). The sequencing of new *Synechococcus* genomes also allowed us to extend the Maestro Microbe hypothesis to *Synechococcus* thermotypes (Zwirglmaier et al., 2008; Pittera et al., 2014), since particularly few genes were found to be specific to the cold-adapted clades I and IV (Supplementary Table S4). In contrast, our analysis of *Synechococcus* core proteins containing amino acid variants shared exclusively by all members of these cold thermotypes revealed potential candidates for adaptation to cold waters (Supplementary Table S9). A number of these core proteins target essential cell functions such as protein metabolism or carbon fixation and metabolism, suggesting that sequence variations of these proteins have an impact on their efficiency at different temperatures. We also identified proteins involved in carotene biosynthesis and the oxidative stress response, suggesting that these pathways are impacted by cold temperature in marine picocyanobacteria. Overall, while experimental testing is needed to validate the role of these substitutions in adaptation to cold environments, this analysis provides numerous strong candidates for such validation (Supplementary Table S9). The fact that all members of clades I and IV share specific variants of the three proteins involved in the β-carotene synthesis pathway (with e.g., >2% of the protein sequence comprising residues specific to these clades in CrtL-b) is particularly striking, since physiological experiments have shown that members of clades I and IV were able to maintain or increase their β-carotene:chlorophyll *a* ratio in response to cold stress, while this ratio decreased in strains representative of warm thermotypes (Pittera et al., 2014). Thus, these substitutions might allow cells of the former clades to maintain β-carotene synthesis in cold conditions, resulting in a reduction of the cold-induced oxidative stress. Additionally, four proteins potentially involved in the response to oxidative stress were found to display variants specific to clades I and IV (Supplementary Table S9). In much the same way, a recent study identified two substitutions in genes encoding the two subunits of phycocyanin in *Synechococcus* between these cold-adapted clades and the warm-adapted clades II and III, which were also thought to be involved in adaptation to distinct thermal niches: RpcA G-43 and RpcB S-42 in the former clades and RpcA A-43 and RpcB N-42 in the latter (Pittera et al., 2017). It is worth noting that these genes were not detected by the stringent approach used here either because of the absence of the multi-copy *cpcA* gene in the CB0101 genome due to assembly issues or to a single exception among the newly sequenced genomes, the clade I strain PROS-9-1 having an RpcB S-42. Given that clades I and IV have diverged about 425 My ago (confidence interval 308–468 My), the most parsimonious explanations for these many shared substitutions would be either an adaptive convergence or an ancient homologous recombination between ancestors of these clades. In this context, it is interesting to note that mutations were found to arise in just a few generations in a clonal *Prochlorococcus* strain as an adaptation to selective conditions such as UV radiation (Osburne et al., 2010), antibiotics (Osburne et al., 2011) or phage pressure (Avrani et al., 2011), emphasizing the role of such substitutions in short-term adaptation, although only a subset of these are kept in the long term.

## Conclusion

Current clades of marine picocyanobacteria might be considered as survivors of a former set of “backbone” populations [as defined by Kashtan et al. (2014)] that appeared hundreds of millions years ago, and then optimized their sequence, while eventually losing most of the genes that initially allowed niche colonization (Lawrence, 2002; Cohan and Koeppel, 2008; Polz et al., 2013; Kashtan et al., 2014). More recently, each of these clades further diversified into a number of new backbone populations, which correspond to the within-clade microdiversity recently described in *Prochlorococcus* and *Synechococcus* (see e.g., Martiny et al., 2009b; Kashtan et al., 2014; Farrant et al., 2016; Larkin et al., 2016). One explanation for the topology of the phylogenetic tree based on core proteins (short branches at the leaves of the tree and long branches at the base of clades, Figure 8) would be the occurrence of periods of rapid diversification, as previously suggested for the occurrence of the different *Synechococcus* clades within SC 5.1 and of the *Prochlorococcus* radiation (Urbach et al., 1998; Dufresne et al., 2008) and more extended periods during which each population stays relatively genetically homogeneous (e.g., by homologous recombination or by frequent genomic sweeps). Alternatively, and perhaps more likely, picocyanobacterial populations might undergo continuous diversification at a fairly constant rate, with diversity purged during rare but severe extinction events, leaving traces only of the surviving ones. While it is tempting to relate these events (diversification or purge) to past geological and climatic shifts, this would need a more thorough examination with an improved time calibration.

One of the next challenges will be to more precisely relate variants (genes or substitutions) to a particular niche. We could advocate achieving this via comparative genomics, but this usually necessitates hundreds to thousands of closely related genomes (for review see Read and Massey, 2014; Chen and Shapiro, 2015), as well as a refined phenotypic characterization of the sequenced strains. Alternatively, one could search *in situ* data for genes or substitutions related to a particular niche or environmental parameter (see e.g., Kent et al., 2016; Grébert et al., 2018; Ahlgren et al., 2019; Garcia et al., 2020). Given the wealth of marine metagenomes that are becoming available for a large variety of environmental niches, such an approach should be particularly powerful to unveil niche adaptation processes in the forthcoming years.

## Materials and Methods

### Genome Sequencing and Assembly

Thirty-four *Synechococcus* strains were chosen for genome sequencing based on their phylogenetic position, pigment content and isolation sites (Figure 1 and Supplementary Table S1). All but the three KORDI strains were retrieved from the Roscoff Culture Collection (RCC^2^) and transferred three times on 0.3% SeaPlaque Agarose (Lonza, Switzerland) to clone them and reduce contamination by heterotrophic bacteria. A first set of 25 *Synechococcus* genomes (including WH8103) were generated at the Genoscope (CEA, Paris-Saclay, France) by shotgun sequencing of two libraries: a short-insert forward-reverse pair-end (PE) library (50–150 bp) and a long-insert reverse-forward mate-pair library (4–10 kb), both sequenced by Illumina^TM^ technology. Additionally, seven other genomes were sequenced at the Center for Genomic Research (University of Liverpool, United Kingdom) by shotgun sequencing of 250 bp reads. Single or PE reads were first assembled into contigs using the CLC Assembly Cell© 4.10 (CLC Bio, Aarhus, Denmark). *Synechococcus* contigs were identified based on their different coverage compared to heterotrophic bacteria, scaffolded using WiseScaffolder and 28 out of 31 genomes were closed by manual finishing as described in Farrant et al. (2015). Three genomes (BIOS-E4-1, BOUM118, and RS9915), had only one to three gaps in highly repeated genomic regions. The base numbering of the circularized genomes was set at 174 bp before the *dnaN* start, corresponding approximately to the origin of replication. Automatic structural and functional annotation of the genomes was then realized using the Institute of Genome Science (IGS) Annotation Engine^3^ (Galens et al., 2011). As concerns KORDI-49, KORDI-52 and KORDI-100 strains, genomes were sequenced from axenic cultures using a 454 GS-FLX Titanium sequencing system (Roche) at Macrogen (Seoul, South Korea). The obtained reads were assembled using the Newbler assembler (version 2.3, Roche). To fill contig gaps, additional PCR and primer walking was conducted. Sequences of all new *Synechococcus* genomes were deposited in GenBank under accession numbers CP047931-CP047961 (BioProject PRJNA596899), except *Synechococcus* sp. WH8103 that was previously deposited to illustrate the performance of the pipeline used to assemble, scaffold and manually finish these genomes as well as the three KORDI genomes that have been deposited in Genbank in August 2014 (see accession numbers in Supplementary Table S1).

### Clustering of Orthologous Genes

Protein and RNA sequences retrieved from new genomes were clustered with genomes previously available (Supplementary Table S1) into CLOGs using the OrthoMCL algorithm (Li et al., 2003) and were then imported into the custom-designed Cyanorak v2.1 information system^4^ for further manual curation and functional annotation. Clustering into CLOGs allowed us to build phyletic patterns (i.e., the number of copies of each gene in each genome per CLOG), which was used to extract lists of genes shared at different taxonomic levels. Core genomes were defined at the genus, sub-cluster and clade levels when more than three genomes were available for a given taxonomic level (see Supplementary Table S2).

The phyletic pattern was also used to estimate the size of the pan-genome and core genome. The sampling of genome combinations necessary to draw pan-genome curves was performed with the software PanGP (Zhao et al., 2014) using as parameters ‘Totally Random,’ SR = 100 and SS = 1000. Pan-genome curves were then drawn with R custom designed scripts (v3.3.1.; R Core Team, 2013). The results of PanGP exponential fits were used as estimates of the asymptotic number of core genes.

### ANI/AAI Calculation

Whole-genome ANI and percentage of conserved DNA between pairs of genomes (percentage of the genome length aligned by Blast with more than 90% ID) were calculated following the method described in Goris et al. (2007). AAI was calculated following the method described by Konstantinidis and Tiedje (2005b). When AAI values differed for a given pair of strains depending on which strain was used as a query for BLAST, the highest value was kept.

### Phylogeny and Tree Comparisons

The *petB* phylogenetic tree was built using PhyML 3.1 (Guindon and Gascuel, 2003) with the HKY model and by estimating gamma parameters and the proportion of invariant sites, based on a database of 230 *petB* sequences (Mazard et al., 2012; Farrant et al., 2016). The confidence of branch points was determined by performing bootstrap analyses, including 1000 replicate data sets. Phylogenetic trees were edited using the Archaeopteryx v0.9901 beta program (Han and Zmasek, 2009). The tree was drawn using iTOL^5^(Letunic and Bork, 2016). Additionally, a set of 821 single-copy core proteins were aligned with MAFFT v7.164b (Katoh and Standley, 2014) and concatenated into a single alignment, resulting in a total of 226,778 amino acids. A phylogenetic tree was built with PhyML 3.1 with the WAG model and estimation of parameters of the gamma distribution and of the proportion of invariant sites. The phylogeny based on gene content was performed as described in Wolf et al. (2002): a Jaccard distance matrix was computed from the phyletic pattern with the package *vegan* (Oksanen et al., 2015) and the matrix was then used by the Neighbor-Joining algorithm implemented in the R package *ape* (Paradis et al., 2004) to generate a tree with 100 bootstraps.

The phylogenetic tree based on core proteins was then compared to the tree based on the phyletic pattern using the R package *dendextend* v.1.3.0 (Galili, 2015). Branch lengths were compared using custom python scripts based on the ete2 toolkit (Huerta-Cepas et al., 2010). Briefly, for each node at the base of a clade (highlighted by blue dots in Figure 8), the average distance from the node to the descending leaves (‘external’ length) and the distance to the parent node (‘internal’ length) were calculated. Boxplots of the distribution of ratios of external to internal branch lengths were drawn in R for both trees and a paired Mann–Whitney–Wilcoxon test assessed the difference between the mean ratios.

### Estimation of Gene Gains and Losses

The number of gene gains and losses were assessed from phyletic patterns using the software Count (Csurös, 2010) that implements a Maximum Likelihood method for estimating the ancestral states (presence, absence, or multiple copies) of every CLOG in the dataset using the phylogenetic core protein tree as reference and allowing four categories for the gamma distribution of duplications and branch lengths (options -transfer_k 1 -length_k 4 -loss_k 1 -duplication_k 4). Cut-off on posterior probability was set at 90%, which allowed us to obtain 2,921 CLOGs at the root of the tree, a number similar to the average number of CLOGs in present-day *Synechococcus* strains. The state of presence-absence of each gene family was then extracted at each node of the tree, and used to calculate the number of gene gains and losses on every branch.

These estimations of gained genes were also used to predict genomic islands in each strain. A genomic island, starting and finishing with full-length gained genes, was defined from consecutive sliding windows (size 10,000 bp, interval 100 bp) with a ratio of nucleotides from gained CDS to total coding nucleotides higher than 50%. A network approach was then applied on all predicted islands to compare the gene content of these islands between all strains. Jaccard distances based on shared gene content were calculated between islands and an edge was drawn to connect two islands if their distance was higher than 0.1 (i.e., when two islands shared at least 10% of their pooled gene content). Network modules detection was then performed using the modularity algorithm (Blondel et al., 2008; resolution = 0.2) implemented in Gephi version 0.9.2 (Bastian et al., 2009). Furthermore, in order to take into account the phylogenetic relatedness between strains sharing genomic islands, a distance matrix based on core protein sequences was computed and used to color edges between nodes. Networks were then represented following the “Atlas 2” spatialization implemented in Gephi.

### Time Calibration of the Tree

The core protein phylogeny was used as input for the *reltime* algorithm (Tamura et al., 2012) and the JTT matrix-based model (Jones et al., 1992), as implemented in MEGA7 (Kumar et al., 2016), with default parameters and SC 5.3 designated as an outgroup. Two calibration points were used, based on Sánchez-Baracaldo (2015) and TimeTree (Kumar et al., 2017): the first calibration point was set on node n2 (Supplementary Figure S7), i.e., the common ancestor of strains WH5701 and WH8102 estimated to have occurred between 582 and 878 My ago, and the second on node n4 (i.e., the common ancestor of strains CC9311 and WH8102; Supplementary Figure S7), set between 252 and 486 My. This method allowed us to relate gain/loss events with the time elapsed on each branch of the tree, taking into account the higher evolution rate of protein-coding genes in *Prochlorococcus* than in *Synechococcus* (Dufresne et al., 2005). We also calculated the number of substitutions for each branch of the tree by multiplying branch length by the total number of residues in the alignment, and divided it by the time elapsed and the branch to obtain a substitution rate per My.

### Estimation of the Number of Fixed Genes and Fixed Substitutions Specific to a Taxon or Shared Between Taxa

At a given node of the tree, genes that were found in all descending leaves and no other strain in the dataset were considered as fixed genes specific to this node. Similarly, every position that showed the same amino acid variant in all leaves below a node and another amino-acid in every other strain were considered as fixed variants specific to this node. Terminal branches were not taken into account in these calculations since, by definition, strain-specific amino acids or genes occurring in these branches cannot be considered as fixed.

Additionally, we also looked in *Synechococcus*-*Cyanobium* core genes for amino acid variants specific to a set of strains corresponding to clades (Supplementary Table S9). A variant was considered as specific to a set of strains if it showed the same amino acid in every strain within the set and any other amino acid in every other strain. To allow comparison between proteins of different lengths, the number of specific variants was normalized by gene length. Given that older clades are expected to have accumulated more substitutions, each set of strains proteins were ranked according to their proportion of specific variants. To identify candidate proteins potentially involved in adaptation to cold conditions in clades I and IV, we took the ratio of the protein rank for the “clades I and IV” set of strains to the median rank for other clades (excluding the clades containing a single strain). We kept only proteins for which this ratio was below 0.33, i.e., proteins with a rank 3 times higher in the “clades I and IV” set than in other clades (Supplementary Table S9).

## Data Availability Statement

The datasets generated in this study can be found in online repositories. The names of the repository/repositories and accession number(s) can be found in the article/ Supplementary Material.

## Author Contributions

FH, MR, FDP, MO, DC, JN, and CS purified newly sequenced *Synechococcus* strains. FH, MR, FDP, DC, JN, and MO extracted the DNA. KL, J-MA, PW, FDP, DC, JN, FP, LG, MH, and GF participated in sequencing and/or assembly of the genomes. MH, GL, EC, AB, LB-G, and GF developed and ran the automatic clustering and annotation pipelines. HD, UG, FP, and LG participated in the expert manual annotation of the genomes. HD, GF, UG, and LG generated and processed the data. UG, HD, JH, and DE produced the genomic island networks. HD, GF, UG, DE, DS, FP, and LG analyzed the results. HD, UG, JH, GF, and LG made the figures. All authors contributed to the preparation of the manuscript. HD, LG, FP, and DS wrote the manuscript. All authors read and approved the final manuscript.

## Conflict of Interest

The authors declare that the research was conducted in the absence of any commercial or financial relationships that could be construed as a potential conflict of interest.

## Footnotes

1 www.sb-roscoff.fr/cyanorak

2 http://roscoff-culture-collection.org/

3 http://ae.igs.umaryland.edu/cgi/index.cgi

4 www.sb-roscoff.fr/cyanorak
