# Supplementary material for: Evolutionary Mechanisms of Long-Term Genome Diversification Associated With Niche Partitioning in Marine Picocyanobacteria
Source: Front Microbiol. 2020 Sep 15;11:567431. doi: 10.3389/fmicb.2020.567431 (PMC7522525; doi:10.3389/fmicb.2020.567431)
Supplement: Supplementary file 2 [file Table_2.DOCX]

Supplementary Material

# Supplementary Figures and Tables

## Supplementary Figures

**Supplementary Figure S1: Relationship between Average Amino-acid Identity (AAI) and Average Nucleotide Identity (ANI).** ANI and AAI are shown in Fig 3A.

**Supplementary Figure S2: Number of gained genes located in genomic islands for all 81 picocyanobacterial genomes.** The color scale indicates the total number of gained genes (log_2_) predicted to be located in genomic islands in each pair of genomes. The diagonal color is thus representative of the number of gained genes in genomic islands in each genome. Strains are ordered according to their phylogenetic relatedness.

**Supplementary Figure S3: Comparison of the genomic islands delineated in previous and current work for a selection of picocyanobacterial strains.** Results are shown for 2 *Prochlorococcus* strains (MED4, HLI and MIT9312, HLII) and 2 *Synechococcus* strains (CC9605, clade II and WH8102, clade III) for which islands were defined in previous studies. The green line indicates the percentage of gained genes in 10 kb windows with a 100 bp step. The black line indicates the 50% cut-off that we applied to delineate genomic islands. The location of islands defined in this study are indicated in orange. The location of islands previously defined in Supplementary Table S3 of [52] and Supplementary Material 5 of [23] are indicated in blue and red, respectively. Abbreviations: ISL and SVR correspond to ‘islands’ and ‘smaller variable regions’, respectively as defined in previous work; GI, genomic islands, as defined in the present work.

**Supplementary Figure S4: Comparison of within and between clades evolution rates.** The boxplots show the distribution of ratios of clade external to internal branch lengths for each node highlighted by blue dots in Fig. 8, as calculated from trees based on core proteins and phyletic patterns, respectively. Differences between the mean ratios were assessed by a paired Mann-Whitney-Wilcoxon test (p-value ≤ 0.0009).

**Supplementary Figure S5: Linear regressions used to calculate the rates of gene gains and the rates of fixation of specific genes. (A)** Maximum-likelihood tree, only the topology is given. Nodes used to calculate evolutionary rates are colored in blue (SC 5.1) and orange (*Prochlorococcus* HL). Circles indicate internal nodes, and squares indicate leaves. **(B)** The rate of fixation of specific genes is calculated as the slope of the linear regression between the number of specific genes and the time elapsed on the leading branch, for internal nodes of SC 5.1 (blue) and HL (orange). **(C)** A zoom on the black rectangle drawn in panel B. **(D)** The rate of gene gains is calculated as the slope of the linear regression between the number of gained genes per node and the time elapsed on the leading branch, for internal nodes (circles, left panel) and leaves (squares, right panel) of SC 5.1 (blue) and HL (orange). **(E)** A zoom on the black rectangle drawn in panel D. Equations and R² are indicated for each regression.

**Supplementary Figure S6: Linear regressions used to calculate the rates of substitution and the rates of fixation of specific substitutions. (A)** Maximum-likelihood tree, only the topology is given. Nodes used to calculate evolutionary rates are colored in blue (SC 5.1) and orange (*Prochlorococcus* HL). Circles indicate internal nodes, and squares indicate leaves. **(B)** The rate of specific amino-acid fixation is calculated as the slope of the linear regression between the number of node-specific amino-acid substitutions and the time elapsed on the leading branch, for internal nodes of SC 5.1 (blue) and HL (orange). **(C)** A zoom on the black rectangle drawn in panel B. **(D)** The rate of amino-acid substitution is calculated as the slope of the linear regression between the number of amino-acid substitutions and the time elapsed on the leading branch, for internal nodes (circles) and leaves (squares) of SC 5.1 (blue) and HL (orange). **(E)** A zoom on the black rectangle drawn in panel D. Equations and R² are indicated for each regression.

**Supplementary Figure S7: Phylogenetic tree of the 81 picocyanobacterial strains based on 821 concatenated core proteins, with internal nodes named.** Maximum-likelihood tree, only the topology is given. Node names used in the text are indicated.

## Supplementary Tables

**Supplementary Table S1: Accession numbers and characteristics of the genomes used in this study.**

**Supplementary Table S2: Core and accessory genes at each taxonomic level (phylum, genus, sub-cluster or clade) corresponding to colored boxes in Fig. 4A.** A CLOG is considered as core in a taxonomic group if it is present in >= 90% of the strains within this group and only taxonomic groups with more than 3 genomes are considered. Accessory genes are shared by at least 2 strains but are not core at any higher taxonomic level. Unique genes are present only in one strain. Paralogs are considered separately.

**Supplementary Table S3: Genes specific to each *Synechococcus* clade.** As only a few genes were found to be strictly clade specific, relaxed rules were used (i.e., genes present in at least 80% of strains within a clade and no other *Synechococcus* strain, cf. column B). Clades gathering only one sequenced strain were not considered (i.e., clades IX, XX and UC-A). CK numbers, i.e. CLOG numbers in the Cyanorak v2.1 database, which are cited in the text are highlighted.

**Supplementary Table S4: Genes specific to sets of *Synechococcus* clades representative of ecologically significant taxonomic unit (ESTU) co-occurring in the field** [21]**.** CLOGs present in at least 90% of strains within a given assemblage and in less than 10% of strains outside of this set (90-10) were selected. When this yielded less than 10 CLOGs, these cut-offs were lowered to more than 80% of strains within a set and less than 20% outside (cf. column B). These relaxed rules allowed us to overcome possible issues resulting from the clustering of orthologous genes and to take into account clades for which the ecological niche is poorly known. CK numbers, i.e. CLOG numbers in the Cyanorak v2.1 database, which are cited in the text are highlighted.

**Supplementary Table S5: Coordinates and composition of every genomic island detected in our dataset.** The description of each CLOG (CK_XXXXXXXX) is available in Supplementary Table 6 for *Prochlorococcus* and Supplementary Table 7 for *Synechococcus*.

**Supplementary Table S6: Composition of modules of *Prochlorococcus* genomic islands.** The strain genomic islands comprised in a module are indicated. Numbers in the table indicate the number of copies of a CLOG that were detected as part of an island of this module. Modules cited in the text and in Fig. 6 are highlighted.

**Supplementary Table S7: Composition of modules of *Synechococcus* genomic islands.** The strain genomic islands comprised in a module are indicated. Numbers in the table indicate the number of copies of a CLOG that were detected as part of an island of this module. Modules cited in the text and in Fig 7 are highlighted.

**Supplementary Table S8: Phyletic pattern of genes involved in phosphorus transport and assimilation in *Synechococcus* and *Cyanobium* spp.** Numbers in the table indicate the number of copies of a CLOG in a given strain unless specified otherwise. The total number of genes per category is also indicated when relevant.

**Supplementary Table S9: Proportion of clade-specific amino acid variants observed in every *Synechococcus*/*Cyanobium* core protein.** For each clade and CLOG, the percentage of specific variants is shown, followed by the CLOG rank based on this percentage. The percentage of specific variants is also indicated for sets of strains corresponding to ESTUs co-occurring in the field (as defined by [21]). CLOGs are ordered according to the ratio of the rank observed in the “clades I/IV set" to the median rank of other clades. Note that clades indicated by a star (light grey) contain a single sequenced strain or two quasi-identical strains (e.g. in the case of SC 5.3) and were not considered in the median calculation. CLOGs selected for the “clade I/IV set" are highlighted in yellow (see main text). Gene names cited in the text are highlighted in green.
