# Supplementary material for: Evolutionary Mechanisms of Long-Term Genome Diversification Associated With Niche Partitioning in Marine Picocyanobacteria
Source: Front Microbiol. 2020 Sep 15;11:567431. doi: 10.3389/fmicb.2020.567431 (PMC7522525; doi:10.3389/fmicb.2020.567431)
Supplement: Supplementary file 3 [file Data_Sheet_1.PDF]

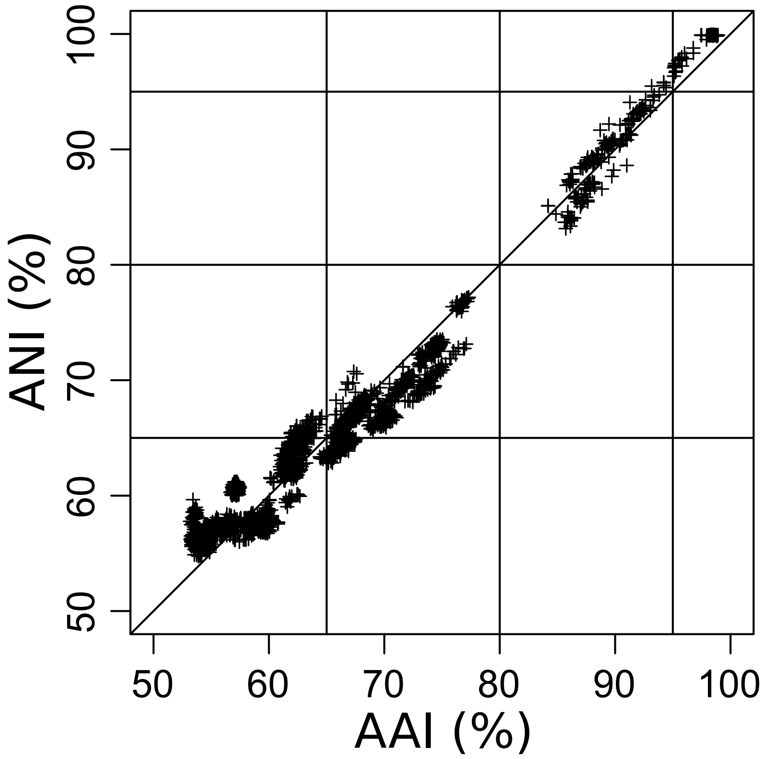

Supplementary Fig. S1: Relationship between Average Amino-acid Identity (AAI) and Average Nucleotide Identity (ANI). ANI and AAI are shown in Fig 3A.

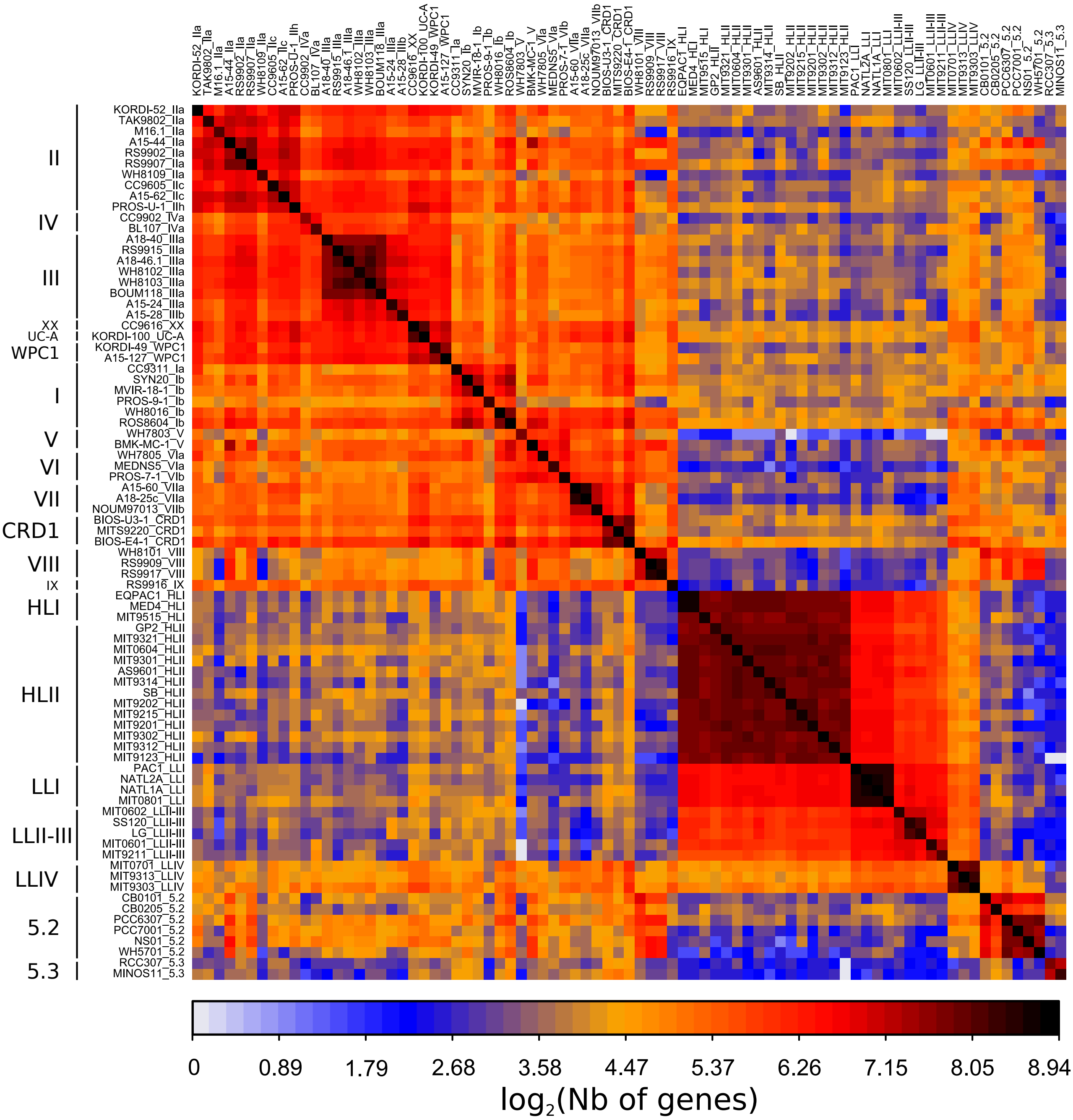

Supplementary Fig. S2: Number of gained genes located in genomic islands for all 81 picocyanobacterial genomes. The color scale indicates the total number of gained genes (log_2) predicted to be located in genomic islands in each pair of genomes. The diagonal color is thus representative of the number of gained genes in genomic islands in each genome. Strains are ordered according to their phylogenetic relatedness.

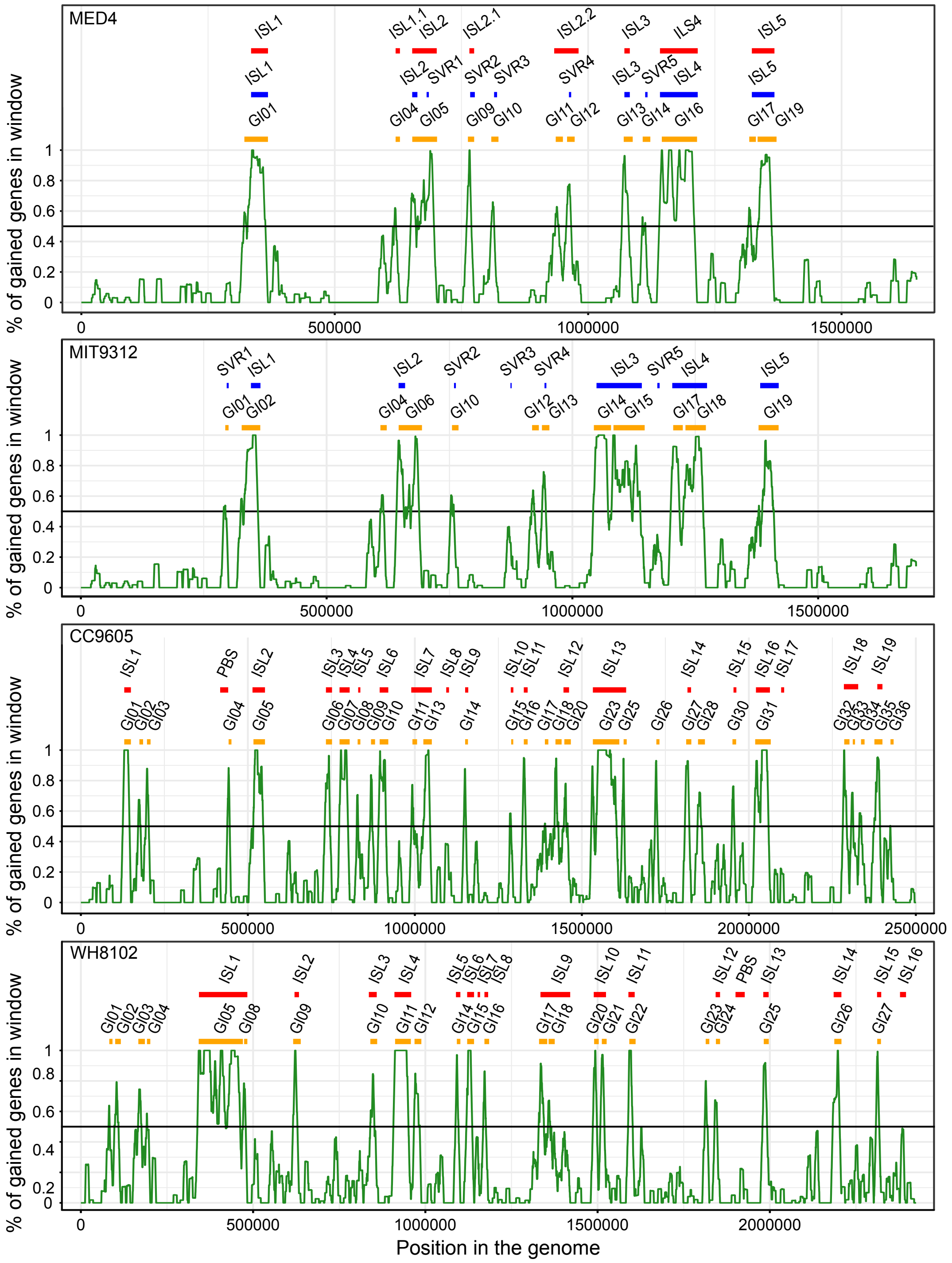

Supplementary Fig. S3: Comparison of the genomic islands delineated in previous and current work for a selection of picocyanobacterial strains. Results are shown for 2 *Prochlorococcus* strains (MED4, HLI and MIT9312, HLII) and 2 *Synechococcus* strains (CC9605, clade II and WH8102, clade III) for which islands were defined in previous studies. The green line indicates the percentage of gained genes in 10 kb windows with a 100 bp step. The black line indicates the 50% cut-off that we applied to delineate genomic islands. The location of islands defined in this study are indicated in orange. The location of islands previously defined in Supplementary Table S3 of Coleman *et al.*, 2006 and Supplementary Material 5 of Dufresne *et al.*, 2008 are indicated in blue and red, respectively. Abbreviations: ISL and SVR correspond to 'islands' and 'smaller variable regions', respectively as defined in previous work; GI, genomic islands, as defined in the present work.

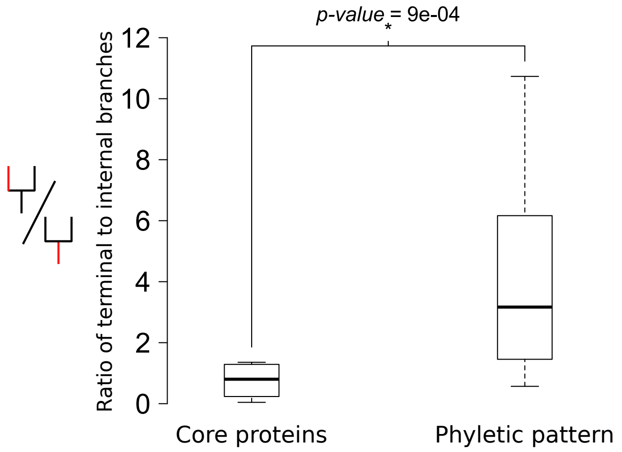

Supplementary Fig. S4: Comparison of within and between clades evolution rates. The boxplots show the distribution of ratios of clade external to internal branch lengths for each node highlighted by blue dots in Fig. 8, as calculated from trees based on core proteins and phyletic patterns, respectively. Differences between the mean ratios were assessed by a paired Mann-Whitney-Wilcoxon test (p-value ≤ 0.0009).

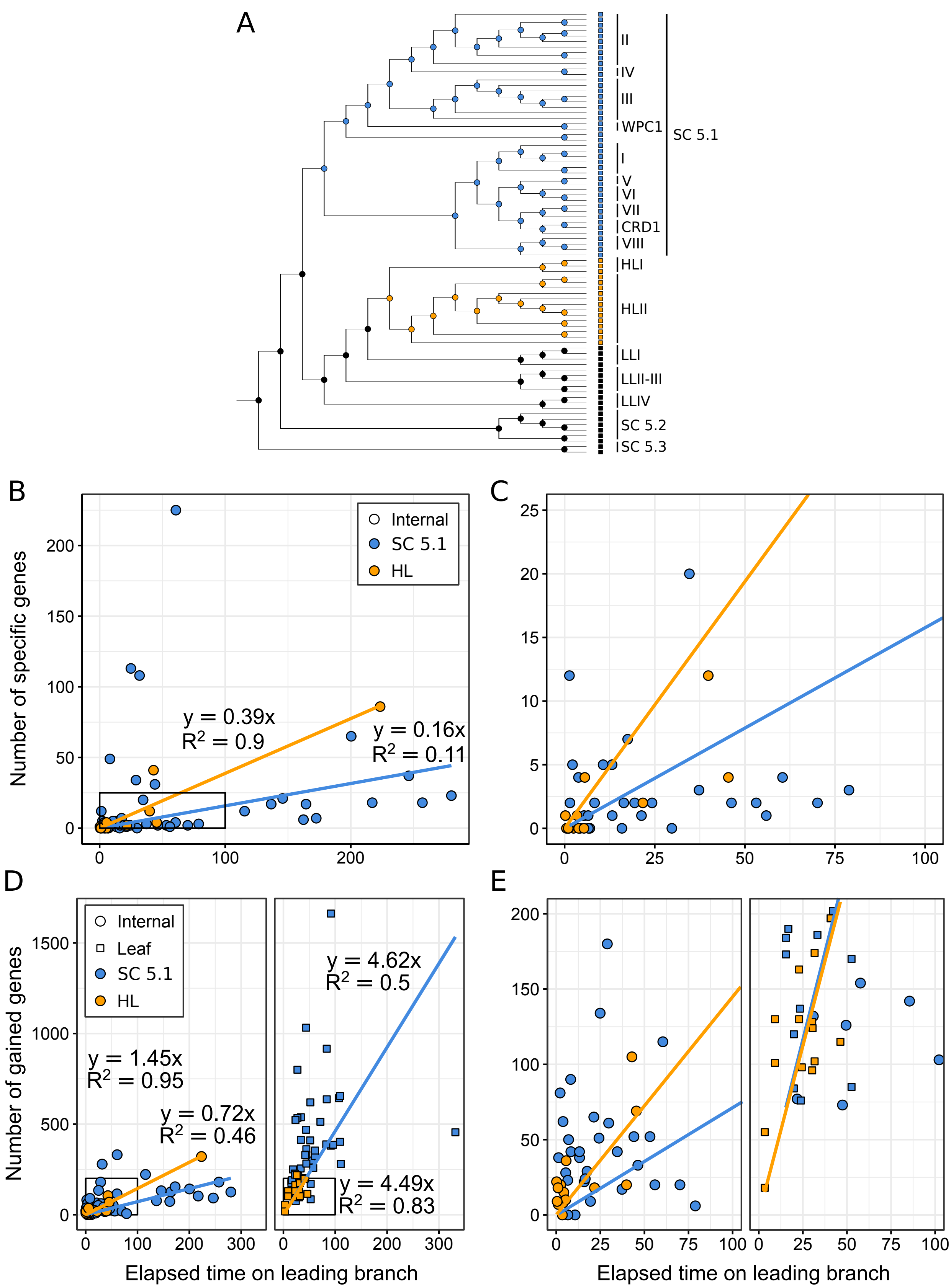

Supplementary Fig. S5: Linear regressions used to calculate the rates of gene gains and the rates of fixation of specific genes. A. Maximum-likelihood tree, only the topology is given. Nodes used to calculate evolutionary rates are colored in blue (SC 5.1) and orange (*Prochlorococcus* HL). Circles indicate internal nodes, and squares indicate leaves. B. The rate of fixation of specific genes is calculated as the slope of the linear regression between the number of specific genes and the time elapsed on the leading branch, for internal nodes of SC 5.1 (blue) and HL (orange). C. A zoom on the black rectangle drawn in panel B. D. The rate of gene gains is calculated as the slope of the linear regression between the number of gained genes per node and the time elapsed on the leading branch, for internal nodes (circles, left panel) and leaves (squares, right panel) of SC 5.1 (blue) and HL (orange). E. A zoom on the black rectangle drawn in panel D. Equations and R^2 are indicated for each regression.

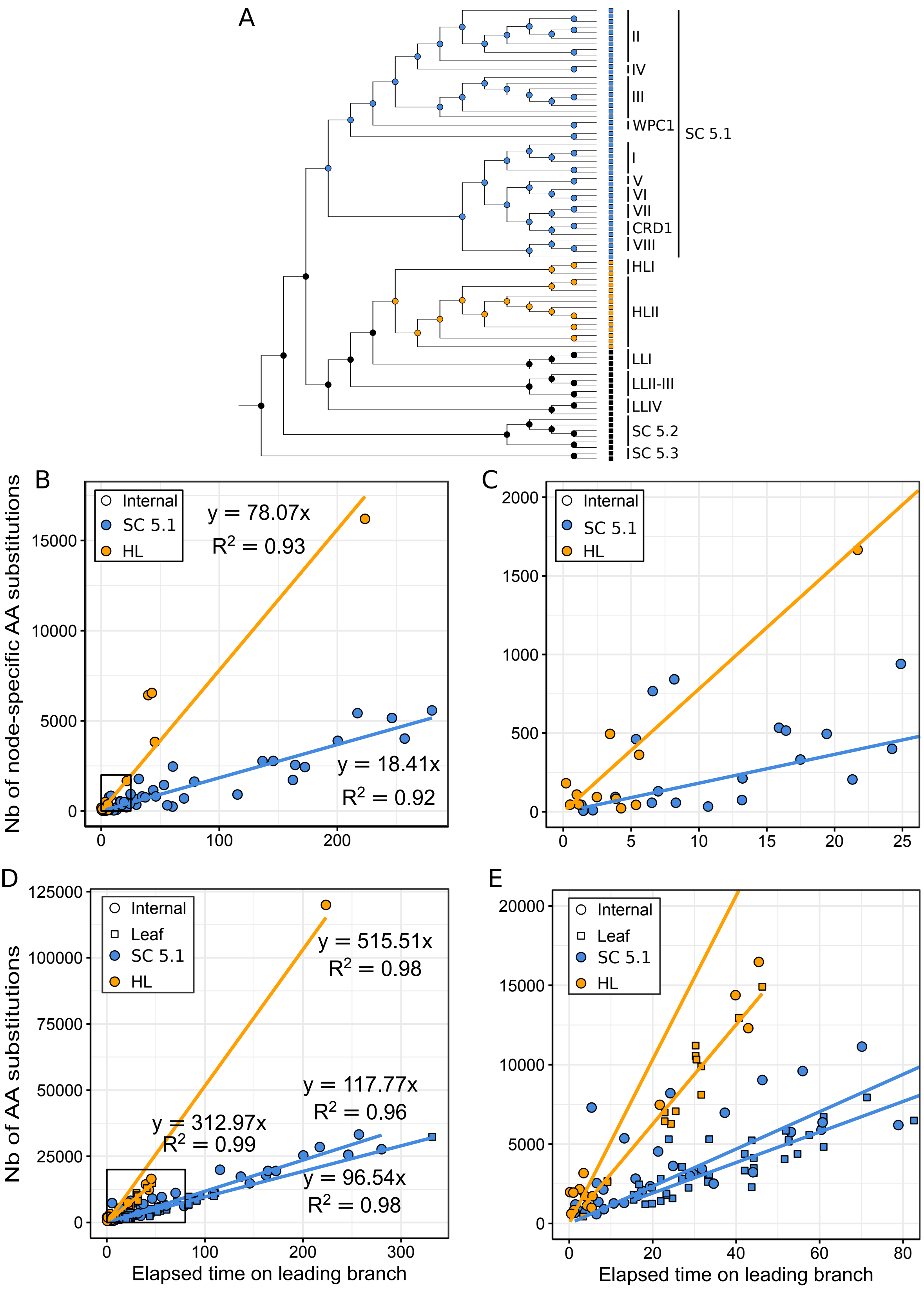

Supplementary Fig. S6: Linear regressions used to calculate the rates of substitution and the rates of fixation of specific substitutions. A. Maximum-likelihood tree, only the topology is given. Nodes used to calculate evolutionary rates are colored in blue (SC 5.1) and orange (Prochlorococcus HL). Circles indicate internal nodes, and squares indicate leaves. B. The rate of specific amino-acid fixation is calculated as the slope of the linear regression between the number of node-specific amino-acid substitutions and the time elapsed on the leading branch, for internal nodes of SC 5.1 (blue) and HL (orange). C. A zoom on the black rectangle drawn in panel B. D. The rate of amino-acid substitution is calculated as the slope of the linear regression between the number of amino-acid substitutions and the time elapsed on the leading branch, for internal nodes (circles) and leaves (squares) of SC 5.1 (blue) and HL (orange). E. A zoom on the black rectangle drawn in panel D. Equations and R² are indicated for each regression.

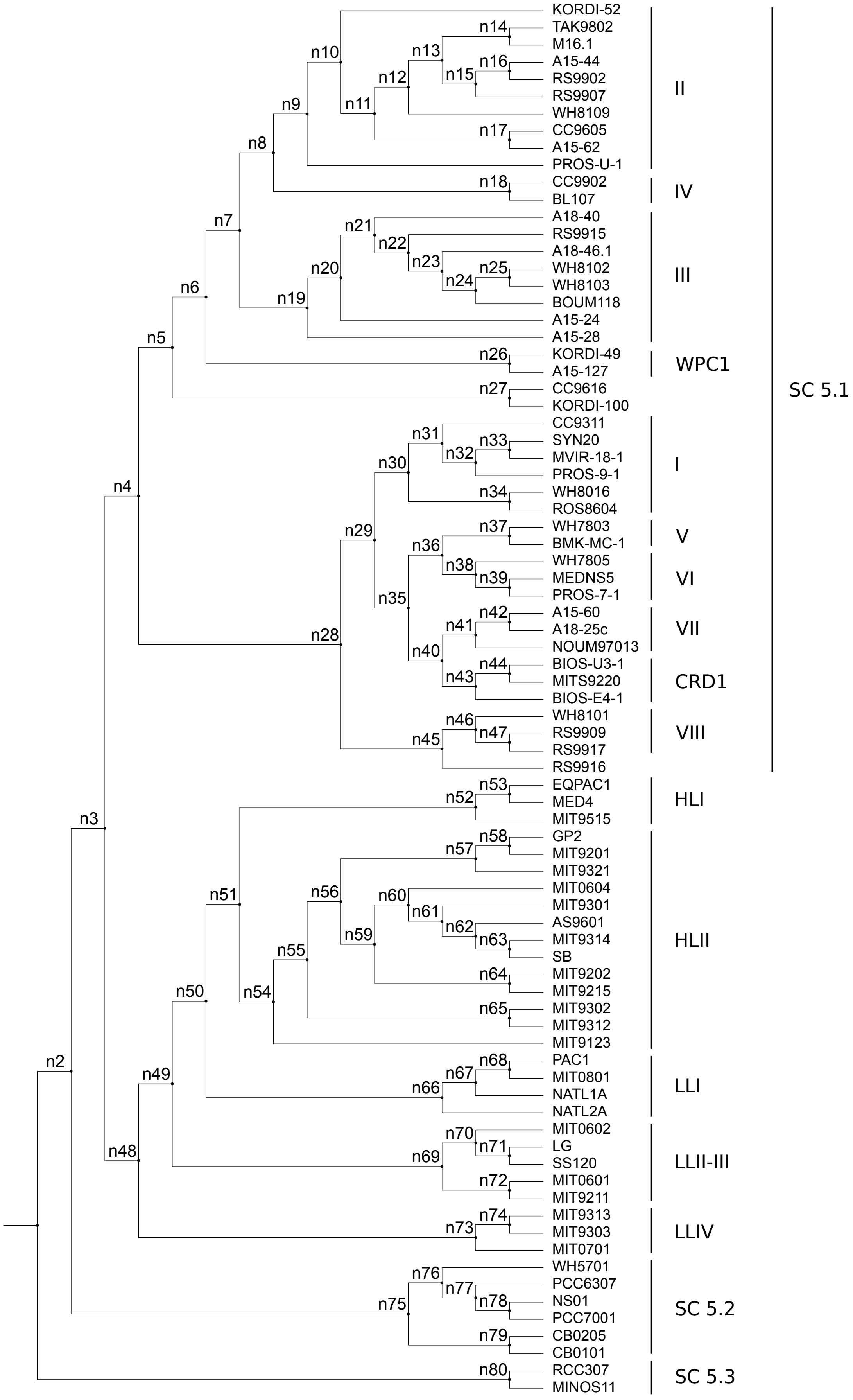

Supplementary Fig. S7: Phylogenetic tree of the 81 picocyanobacterial strains based on 821 concatenated core proteins, with internal nodes named. Maximum-likelihood tree, only the topology is given. Node names used in the text are indicated.
